# Single-cell stabilization method identifies gonadotrope transcriptional dynamics and pituitary cell type heterogeneity

**DOI:** 10.1093/nar/gky991

**Published:** 2018-10-24

**Authors:** Frederique Ruf-Zamojski, Yongchao Ge, Venugopalan Nair, Michel Zamojski, Hanna Pincas, Chirine Toufaily, Jessica Tome-Garcia, Marlon Stoeckius, William Stephenson, Gregory R Smith, Daniel J Bernard, Nadejda M Tsankova, Boris M Hartmann, Miguel Fribourg, Peter Smibert, Harold Swerdlow, Judith L Turgeon, Stuart C Sealfon

**Affiliations:** 1 Department of Neurology, Center for Advanced Research on Diagnostic Assays, Icahn School of Medicine at Mount Sinai, New York, NY 10029, USA; 2 Dept. of Pharmacology and Therapeutics, McGill University, Montreal, Quebec H3G 1Y6, Canada; 3 Department of Pathology, Icahn School of Medicine at Mount Sinai, New York, NY 10029, USA; 4 Department of Neuroscience, Friedman Brain Institute, Icahn School of Medicine at Mount Sinai, New York, NY 10029, USA; 5 Tisch Cancer Institute, Icahn School of Medicine at Mount Sinai, New York, NY 10029, USA; 6 New York Genome Center, New York, NY 10013, USA; 7 Department of Internal Medicine, University of California, Davis, CA 95616, USA

## Abstract

Immediate-early response genes (IEGs) are rapidly and transiently induced following an extracellular signal. Elucidating the IEG response patterns in single cells (SCs) requires assaying large numbers of timed samples at high accuracy while minimizing handling effects. To achieve this, we developed and validated RNA stabilization Buffer for Examination of Single-cell Transcriptomes (RNA-Best), a versatile single-step cell and tissue preservation protocol that stabilizes RNA in intact SCs without perturbing transcription patterns. We characterize for the first time SC heterogeneity in IEG responses to pulsatile gonadotropin-releasing hormone (GnRH) stimuli in pituitary gonadotrope cells. Our study identifies a gene-specific hierarchical pattern of all-or-none transcript induction elicited by increasing concentrations of GnRH. This quantal pattern of gene activation raises the possibility that IEG activation, when accurately resolved at the SC level, may be mediated by gene bits that behave as pure binary switches.

## INTRODUCTION

Immediate-early response genes (IEGs) are rapidly upregulated in response to various external stimuli such as growth factors, hormones, or stress (1,2). IEGs respond to external stimuli within minutes, without requiring *de novo* protein synthesis. Most IEGs encode transcription factors, which regulate genes involved in various cellular functions (3). The quantitative relationship between stimulus dose and transcriptional response is key for an appropriate cell response (4).

IEG induction by hypothalamic gonadotropin-releasing hormone (GnRH) is involved in the regulation of gonadotropin subunit gene (*Lhb* and *Fshb*) expression in pituitary gonadotropes and in gonadotrope cell lines (5–9). The pattern of GnRH signal is pulsatile, which influences gonadotropin gene regulation and is critical for reproductive function (10,11).

Previous studies of IEG transcriptional responses to a stimulus mainly have relied on RNA measurements in bulk cell populations (8,9,12,13). The recent development of SC transcriptomics has enabled researchers to explore cell-to-cell variability and grasp the complex dynamics of gene regulation. Our recent SC transcriptome analysis of gonadotrope cells exposed to static GnRH stimulation highlighted cellular heterogeneity in the response to GnRH and the absence of an effect of cell cycle on this response (14). However, SC sequencing approaches are limited in measurement accuracy due to statistical limitations on measuring rare transcripts. We sought to characterize IEG transcriptional responses to increasing doses of a physiologic pulsatile GnRH stimulus across dynamic time-varying stages using integrated fluidic circuit (IFC) SC real-time PCR assays and via a new method for stabilizing the large number of samples needed for study. We devised SC studies that included detailed GnRH pulse stimulation time course experiments (15) and examination of the gonadotrope response to varying GnRH concentrations (16).

Dissecting immediate-early transcriptional responses to an external stimulus in individual cells requires elaborate study designs that include various treatments, time points, and replicates. IEGs, in particular, are dynamic and can respond to handling (17,18). The use of fresh cells for SC transcriptomics necessitates immediate sample processing, which is incompatible with complex time course studies and with a separation between sample harvest and SC processing steps. Thus, there is a need for a cell preservation protocol that: (i) allows splitting of collection time from processing time, (ii) minimizes cell disturbance and transcriptome changes. Such a protocol would also eliminate an obstacle to SC transcriptome analysis of research or diagnostic samples obtained in clinical settings. Although SC preservation approaches (19–21,22) have been reported, they involve multistep research laboratory handling (centrifugation, resuspension) and may be impractical for processing hundreds of samples needed to accurately resolve transcriptional patterns.

We developed a single-step cell preservation method referred to as RNA stabilization Buffer for Examination of Single-cell Transcriptomes (RNA-Best). RNA-Best minimizes alteration of the cell transcriptional state and is suitable for exploring the temporal dynamics of immediate-early transcriptional responses in a large sample set. We validated this methodology by showing that: (i) it maintains RNA quality and qPCR assay-based expression patterns of IEGs in a mouse gonadotrope cell line, (ii) it preserves bulk transcriptome integrity and expression profiles both in a gonadotrope cell line and in primary pituitary cells, (iii) it is applicable to SC transcriptome studies, including IFC SC qPCR of human and mouse cells and SC RNA-seq of mouse gonadotrope cells. Furthermore, RNA-Best enables SC profiling of primary cultures of mouse pituitaries and of dissociated human brain biopsies. Critically, the use of RNA-Best allowed us to investigate cell-to-cell variability in IEG responses to GnRH pulses across various times and doses. We provide first evidence that IEGs show a binary all-or-none SC response to GnRH, with different genes exhibiting distinct probabilities of induction at each concentration.

## MATERIALS AND METHODS

### Cell culture

GnRH was purchased from Bachem (Torrance, CA, USA). LβT2 cells were obtained from Dr. Pamela Mellon (University of California, San Diego, CA). Cells were cultured at 37°C in DMEM (Mediatech, Herndon, VA) supplemented with 10% fetal bovine serum (FBS; Gemini, Calabasas, CA, USA) in a humidified air atmosphere of 5% CO2. Cells were regularly tested (every 3–6 months) for mycoplasma and interspecies contamination, and authenticated by analysis of short tandem repeat (STR) DNA profiling using 27 mouse-specific microsatellite markers (Mouse Cell Check Plus, IDEXX BioResearch, Columbia, MO, USA). Cell line authentication was achieved by comparing our cells with an original aliquot of LβT2 cells used as a standard reference. Our results confirmed that our LβT2 cells were mycoplasma-free, were of mouse origin, and had similar markers as the original cell line aliquot.

### Cell preparation and RNA extraction for cell populations

#### Cell preparation and preservation

LβT2 cells were seeded at 350 000 cells per well in 12-well plates in DMEM/10% FBS. After 2 days of culture at 37°C/5% CO_2_, cells were treated with either vehicle (i.e. medium only) or GnRH (2 nM) for various time periods. Cells were then trypsinized, pelleted at 2200 rpm for 5 min, and resuspended in 400 μl RNA-Best). RNA-Best-preserved cells were stored at 4°C in 1.5 ml Eppendorf tubes for at least 4 days, then spun down at 2200 rpm for 5 min. Pelleted cells were then lysed in 360 μl guanidium thiocyanate RNA lysis buffer. The proprietary RNA-Best reagent is available upon request from the authors.

To compare RNA-Best with the use of fresh cells and other existing cell preservation/stabilization methods, we proceeded with the following alternate protocols after cell trypsinization and pelleting: (i) direct cell lysis in a guanidium thiocyanate RNA lysis buffer (4 M guanidium thiocyanate, 25 mM sodium citrate pH 7, 0.5% sarcosyl [N-lauroyl sarcosine] and 0.1 M 2-mercaptoethanol) (23) and storage at −20°C, (ii) cell fixation with 4% PFA and storage at −80°C, as previously described (21), (iii) cell freezing in DMEM supplemented with 20% FBS and 10% DMSO, and storage at −80°C (20) and (iv) methanol fixation and storage at −80°C (19).

#### RNA extraction and quality control

Except for PFA-fixed cells, total RNA was extracted using the ‘Absolutely RNA 96 microprep’ kit (Agilent, Santa Clara, CA, USA) according to the manufacturer's protocol, and resuspended in elution buffer (Agilent, Santa Clara, CA). By contrast, cells fixed with PFA were subjected to reverse-cross-linking and cell lysis, and RNA purification using the miRNeasy FFPE Kit (Qiagen, #217504) (protease lysis, reverse-crosslinking method (PLRC)), as previously described (21). RNA quality and quantity were assessed by spectrophotometry (Nanodrop), fluorometry (Quant-iT RiboGreen RNA reagent, Invitrogen, Carlsbad, CA, #R11490; Qubit RNA High sensitivity or Broad Range Assay Kits, depending on the RNA amount, ThermoFisher Scientific), and on a Bioanalyzer (Agilent RNA Nano and Pico kits). The RNA Integrity Number (RIN) was determined with Bioanalyzer; a RIN of 8 or above indicated that a sample was suitable for further processing.

### Bulk quantitative real-time PCR

qPCR was performed as recently described (14).

### Bulk RNA-seq assay

Cell preparation, RNA-Best preservation, and RNA extraction were as described above, with the exception that cells were merely treated with 2 nM GnRH for 40 min. RNA-seq libraries were prepared with 2 μg of RNA using the Illumina Truseq LT mRNA kit (Illumina, #RS-122-2101). ERCCs (DNA Sequence Library for External RNA, Controls, #2374, National Institute of Standards and Technology) were added for analysis and quality assessment. Library quality control and quantification were assessed by spectrophotometry (Nanodrop), fluorometry (Qubit dsDNA High sensitivity Assay Kit), qPCR (Kapa Library Quantification Kit Illumina Platforms, Kapa Biosystems, #KK4835), and on a Bioanalyzer (High-Sensitivity DNA Bioanalyzer kit, Agilent). Additionally, the quality of each library was assessed by qPCR of selected genes. A total of six libraries (three from fresh cells, three from RNA-Best-stabilized cells) were pooled together at equal concentration, and the pooled sample (20 μl, 10 nM) was sequenced at the Epigenomics Core of Weill Cornell Medical College on Illumina HiSeq 2500 v3 using 101 bp single reads.

### SC stabilization

LβT2 cells were trypsinized, spun down, resuspended in RNA-Best and stored at 4°C until the assay was performed. Samples preserved in RNA-Best at 4°C were stable for at least 8 weeks.

### High-throughput microfluidic SC qPCR

LβT2 cells were seeded on glass coverslips and treated with either 0.5, 2, 8 or 20 nM GnRH pulses (or vehicle) every 2 h for a total of four pulses, as previously described (15). Cells were harvested at short time intervals around the fourth pulse: 1 min before the fourth pulse (–1 min), 25 min after (+25 min), and 35 min after the fourth pulse (+35 min); for GnRH-treated samples, an additional sample was harvested 60 min after the fourth pulse (+60 min). A ‘no pulse’ control sample was also collected to determine the basal level of activation. Coverslips were collected and cells were trypsinized, pelleted, resuspended in 1 ml RNA-Best, and stored at 4°C. Of note, for each experimental condition (i.e. GnRH concentration and time point), three biological replicates were used for bulk qPCR analysis, and one for SC qPCR analysis.

On the day of the Fluidigm integrated fluidic circuit (IFC) C1 experiment, RNA-Best-stabilized cells were counted and diluted to 200 000 cells/ml in RNA-Best, filtered through a 10-μm filter, and resuspended in a 3:2 ratio of RNA-Best:C1 suspension reagent, as recommended by the manufacturer. SCs were isolated using the IFC C1 system. Briefly, the Pre-amplification assay (PN 100-4904 H1) was performed according to the standard Fluidigm protocol using a small 5–10 μm primed chip, and was followed by the STA-Pre-Amp script. SC cDNAs were collected into 25 μl of C1 DNA dilution reagent.

qPCR was performed using a Fluidigm Biomark HD system. A total of 68 markers were analyzed: 54 genes, 3 Fluidigm spikes, 8 ERCC spikes (both RNA and DNA) and 5 DNA markers. Briefly, 10X assays were prepared to obtain a final 10× concentration with 9 μM of primers, 2.5 μM of probe (Roche Universal probes), and assay loading reagent (Fluidigm PN 100-7611). Samples were prepared by mixing together 3 μl of 2× mastermix (Applied Biosystems, Taqman Gene expression Master Mix #4369016), 0.3 μl of 20X GE sample loading reagent (Fluidigm PN 100-7610), and 2.7 μl of pre-amplified cDNA. The 96.96 dynamic array chip was primed, then loaded with 5 μl of 10× assays and 5 μl of sample mix. The plate was loaded into an HX Fluidigm system for the priming and loading steps using the Fluidigm scripts. Upon completion of the loading script, the plate was transferred to a Biomark instrument to collect qPCR data using the Biomark HD Data Collection software. The parameters specified were: ROX (passive reference), single assay Probe (FAM-TAMRA), GE 96.96 standard v1 without melting curve, and Auto-exposure. Data were analyzed using the Fluidigm Real-Time PCR analysis software to generate the heat map and individual well results. The automated function was used to determine Cycle-Threshold (Ct) values, and data were visually inspected to make sure the threshold looked fine. The Ct threshold was manually adjusted in the case of the *Fos* gene at 20 nM GnRH. Data were exported into Excel for further analysis. Gene expression was calculated as 41 – Ct value. Wells that showed no expression of house-keeping genes represented either damaged cells, cell debris, or the absence of cell, and thus were removed from further analysis.

### GEM Drop-seq assay

LβT2 cells were treated with either vehicle or 2 nM GnRH for 40 min. Cells were then trypsinized, pelleted, and resuspended in 1 ml RNA-Best. GEM Drop-seq was performed as described (10× Genomics, Pleasanton, CA, USA; (24)), following the Single Cell 3′ Reagents Kits V2 User Guide. Cells were filtered, counted on a Countess instrument, and the final concentration was set at 1,000 cells/μl in RNA-Best. The 10X chip (Chromium Single Cell 3′ Chip kit v2 PN-12036) was loaded to target 5000 cells final. Reverse-transcription was performed in the emulsion and cDNA was amplified for 12 cycles before library construction (Chromium Single Cell 3′ Library and Gel Bead Kit V2 PN-120237). Each library was tagged with a different index for multiplexing (Chromium i7 Multiplex kit PN-12062). Quality control and quantification of the amplified cDNA were assessed on a Bioanalyzer (High-Sensitivity DNA Bioanalyzer kit). Library quality control and quantification were evaluated as described above. Sequencing was carried out at the Epigenomics Core of Weill Cornell Medical College on Illumina HiSeq 2500 v3 using 98+26 paired-end reads, two lanes, rapid mode.

### Bulk RNA-seq data analysis

RNA-seq reads were aligned using STAR (25) v2.5.1b with the mouse genome (GRCm38 assembly) and gene annotations (release M8, Ensembl version 83) downloaded from https://www.gencodegenes.org/. The matrix counts of gene expression for all six samples were computed by featureCounts v1.5.0-p1 (26). Differentially expressed genes (5% FDR and at least 2log_2_ fold change) were identified using the voom method (27) in the Bioconductor (28) package Limma (29). Pearson correlation was computed in R using the cor() function (30). The TPM computed by RSEM (31) was used for the comparison of bulk RNA-seq with SC RNA-seq data.

### SC RNA-seq data analysis

SC RNA-seq data were processed using the Cell Ranger pipeline v1.3, which provides a data matrix of expression for all genes and all cells. Differentially expressed genes were analyzed using the sSeq method (32), as implemented in the R package cellrangerRkit v1.1. The cell phase computation for the SCs follows the ideas described in the Supplementary Material of Macosko *et al.* (33) with our own customized R script implementation.

### Statistics

**Figure 1.**
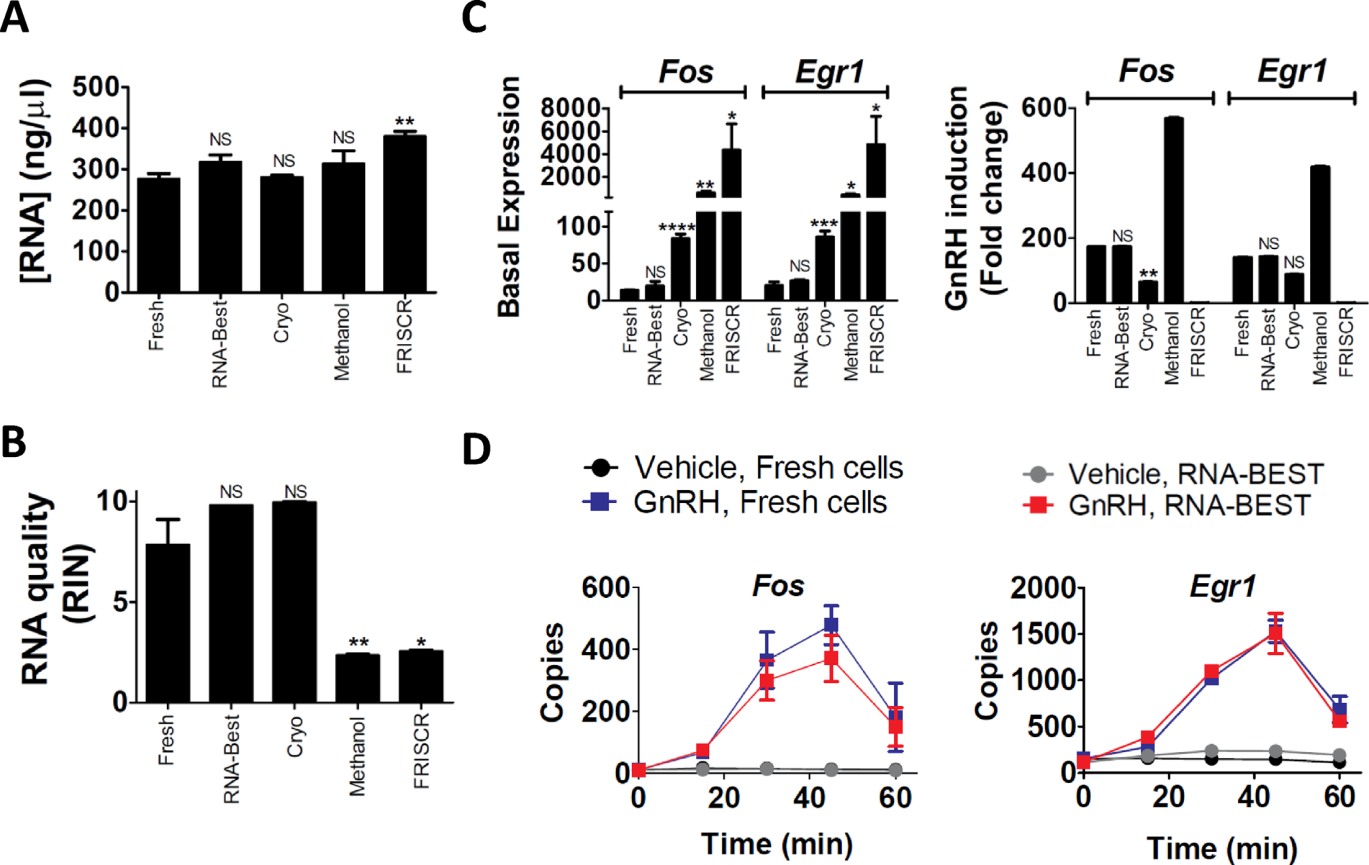
RNA-Best preservation of cultured gonadotropes maintains IEG transcript levels under basal and stimulated conditions. (A, B) RNA yield (**A**) and quality (**B**) from fresh cells, RNA-Best-preserved cells, or cells preserved using cryopreservation, methanol fixation, or FRISCR. *P < 0.05 and **P < 0.01; bars show mean ± s.e.m. (**C**) qPCR analysis of *Fos* and *Egr1* basal expression and GnRH induction (fold change); 2 nM GnRH treatment for 35 min; *P < 0.05, **P < 0.01, ***P < 0.001 and ****P < 0.0001. Note that *Egr1* fold change using FRISCR equals 1, due to no difference between vehicle and GnRH treatment. (**D**) Time course of GnRH induction of *Fos* and *Egr1* in fresh vs. RNA-Best-preserved cells (*n* = 6 biological replicates per time point and protocol). Data in A–D are from one of four representative experiments.

For assessment of the effect of SC preservation on RNA yield (Figure 1A), we used a one-way analysis of variance (ANOVA) followed by Bonferroni multiple comparison post-hoc test, with *n* = 8 biological replicates per protocol and *F* = 5.523. The number of degrees of freedom was 39. For analysis of RNA integrity (Figure 1B), we used one-way ANOVA followed by Bonferroni multiple comparison test, with *n* = 2 biological replicates per protocol and *F* = 45.73. The number of degrees of freedom was 9. For evaluating the effects of preservation on basal and regulated transcript levels by bulk qPCR (Figure 1C), we used a two-tailed *t*-test, with *n* = 4 biological replicates. For *Fos* basal expression, the *t*-values (*t*) and the number of degrees of freedom (df) were: *t* = 1.066, df = 6 (Fresh cells versus RNA-Best preservation), *t* = 10.69, df = 6 (fresh cells versus cryopreservation), *t* = 4.239, df = 6 (fresh cells versus methanol fixation), *t* = 4.322, df = 4 (fresh cells versus FRISCR). For *Egr1* basal expression, they were: *t* = 1.061, df = 5 (fresh cells versus RNA-Best preservation), *t* = 6.715, df = 6 (fresh cells versus cryopreservation), *t* = 3.289, df = 6 (fresh cells versus methanol fixation), *t* = 4.426, df = 4 (fresh cells versus FRISCR). For *Fos* induced expression, they were: *t* = 1.265, df = 6 (fresh cells versus RNA-Best preservation), *t* = 4.650, df = 6 (fresh cells versus cryopreservation). For *Egr1* induced expression, they were: *t* = 1.867, df = 6 (fresh cells versus RNA-Best preservation), *t* = 1.228, df = 6 (fresh cells versus cryopreservation). Statistical analyses were all performed using GraphPad Prism version 5.04 (GraphPad Software, San Diego, CA, USA, www.graphpad.com).

## RESULTS

### Preservation of RNA integrity and qPCR-based IEG expression patterns in RNA-Best-stabilized cells

We compared RNA isolated from LβT2 gonadotrope cells preserved for 10 days at 4°C with RNA-Best, to that of cells stored at −80°C with other SC preservation methods (19–21), and to RNA isolated from fresh cells. We assessed cellular and RNA integrity, gene regulatory alterations caused by preservation, and ease of use. Total RNA yield was fairly similar with all approaches (Figure 1A). The high RNA integrity numbers (RINs) seen in fresh cells were only found with the RNA-Best and cryopreservation protocols (Figure 1B, Supplementary Figure S1). RINs were significantly lower with FRISCR and methanol fixation. To evaluate the effects of preservation on basal and regulated transcript levels, we measured induction of the IEGs *Fos* and *Egr1* by GnRH (9). Only cells preserved with RNA-Best showed basal and regulated transcript expression levels that were not significantly different from those observed in fresh cells (Figure 1C). A detailed time course of *Fos* and *Egr1* induction by GnRH showed that indistinguishable temporal gene expression trajectories were obtained with RNA-Best-preserved and fresh cells (Figure 1D). Altogether, these data suggest that the RNA-Best protocol preserves high quality RNA with negligible alteration of gene expression patterns, even for handling-sensitive transcripts.

### RNA-seq expression profiles of fresh and RNA-Best-preserved bulk cells are comparable

**Figure 2.**
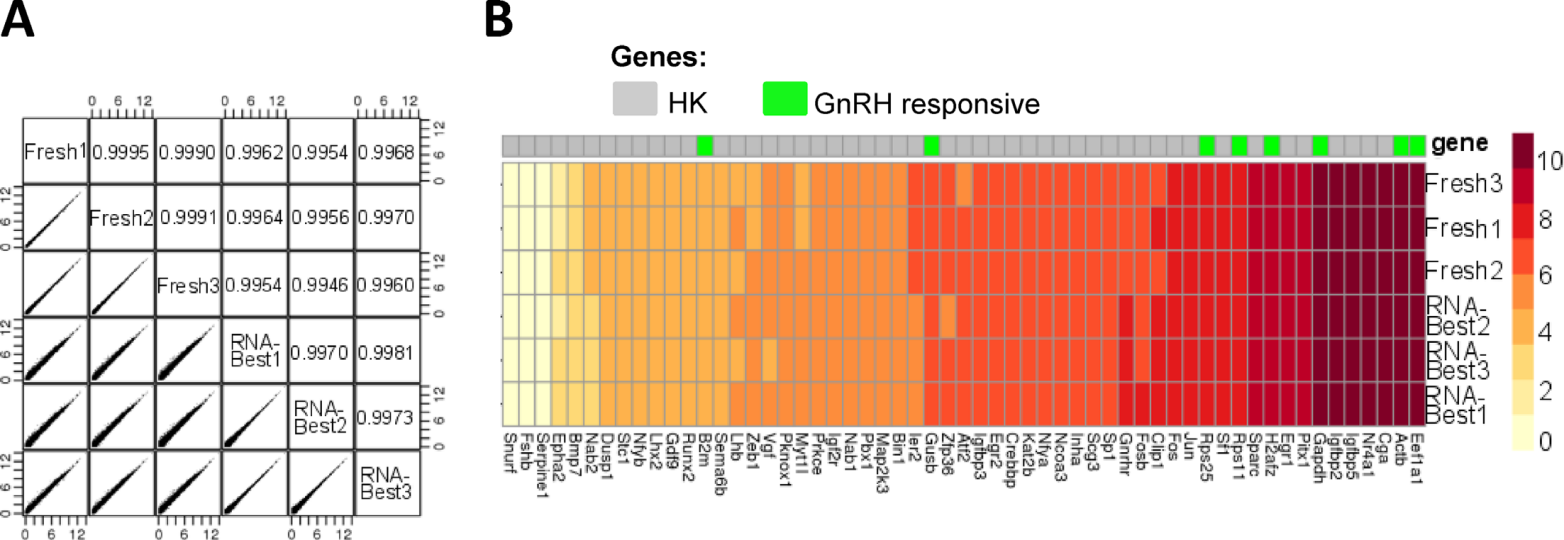
RNA-Best preserves transcriptome profiles from bulk gonadotrope cells. (**A**) Pairwise correlations between bulk RNA-seq data from three fresh and three RNA-Best samples. Expression counts were converted to log_2_ (counts per million +1). Pearson correlations are indicated. (**B**) Heat map of selected GnRH-regulated genes and commonly used house-keeping (HK) genes in the same samples as in A.

To evaluate our approach for the transcriptomic profiling of LβT2 cells, we compared bulk RNA sequencing (RNA-seq) data obtained from RNA-Best-preserved and fresh samples following GnRH treatment. Quality assessment of RNA-seq libraries revealed comparable Bioanalyzer traces (Supplementary Figure S1). Gene expression levels were highly consistent based on pairwise correlations (Figure 2A) and expression heat map (Figure 2B). Sequenced samples were also comparable with respect to chromosomal transcript representation bias and GC coverage (Supplementary Figure S2), as well as 5′ to 3′ read coverage bias across all predicted transcript lengths, with the expected increased bias for longer transcripts (Supplementary Figure S3).

### RNA-Best maintains SC transcriptome integrity

**Figure 3.**
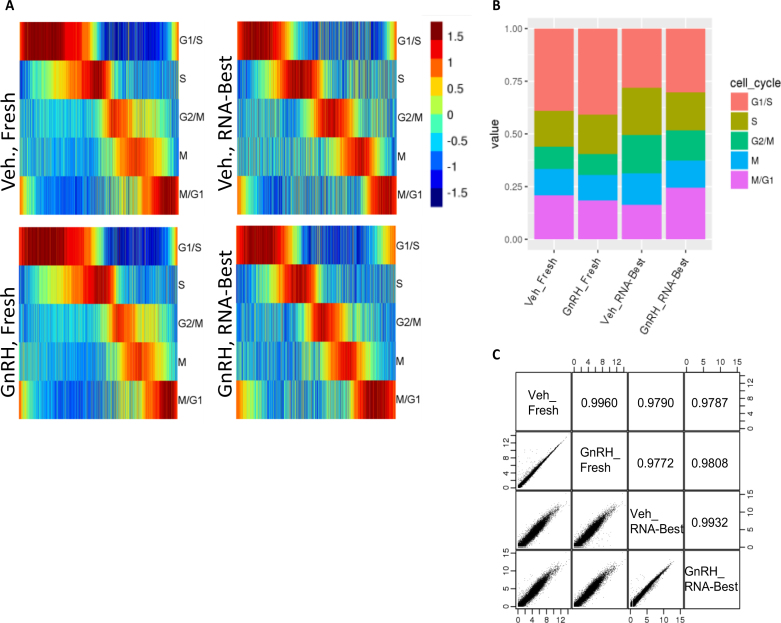
RNA-Best preserves cell cycle stage assignments in single gonadotrope cells exposed to GnRH. (A, B) Cell cycle and GnRH-cell cycle interaction effects on SC transcriptome in fresh (1992 vehicle- and 1889 GnRH-treated) vs. RNA-Best-preserved cells (3579 vehicle- and 2753 GnRH-treated) using GEM Drop-seq. (**A**) Each cell on the x-axis is aligned by cell cycle progression. Color coding indicates score assignment to the cell cycle phase. The five cell cycle phases are indicated. (**B**) Summary of the percentage of cells assigned to different cycles. (**C**) Pairwise correlations between SC gene expression measurements averaged across vehicle- vs. GnRH-treated cells that were either directly lysed (fresh cells) or preserved in RNA-Best. Expression counts were converted to log_2_ (counts per million +1). Pearson correlations are indicated.

We evaluated the use of RNA-Best for studying variation in SC transcriptomes. Imaging flow cytometry analysis of RNA-Best-stabilized LβT2 cells showed that SCs were preserved (Supplementary Figure S4). Analysis of SC transcripts from an RNA-Best-preserved human monocyte and mouse LβT2 cell mixture assayed by integrated fluidic circuit (IFC) qPCR showed a high measurement consistency across SCs and a low cell doublet rate (Supplementary Figure S4). We assessed the use of this protocol for the analysis of cell-to-cell variability in individual cycling cells using gel-in-emulsion (GEM) Drop-seq (24). We recently used GEM Drop-seq in fresh samples of LβT2 cells to determine the SC cell cycle stage assignment and the interaction of cell cycle with the effects of GnRH (14). We reproduced this experiment using cells preserved with RNA-Best. While small differences in the proportion of cells in each cycle were noted, presumably due to the studies being performed on different batches of cells, the findings and conclusions about GnRH-cell cycle interaction effects were equivalent (Figure 3A, B). When comparing averaged SC transcript measurements from fresh vs. RNA-Best-stabilized cells (for all the treatment combinations), correlation coefficients were within the 0.97–0.99 range (Figure 3C), most likely due to the batch effect mentioned above. In comparison, the correlation coefficient for vehicle- versus GnRH-treated cells within each method was >0.99, underscoring data reproducibility.

### RNA-Best enables cell type profiling in mouse and human primary tissues

We evaluated the use of RNA-Best for overcoming geographical and practical barriers through a collaborative SC analysis of primary cultures and acutely dissociated tissues using a variety of SC assay protocols. Primary mouse pituitary cells were isolated in a laboratory separated by 400 miles (and an international border) from the assay laboratory and preserved in different reagents during transport. Primary pituitary samples preserved with RNA-Best or with an established RNA-stabilizing reagent that does not preserve SCs, when analyzed as bulk samples, showed comparable read sequence characteristics and transcript expression patterns (Supplementary Figures S5 and S6). Based on a pairwise correlation and a heat map of highly expressed genes, gene expression levels were highly correlated (Supplementary Figure S5). Analysis of SC heterogeneity in primary mouse pituitary cultures using IFC-SC isolation followed by SC RNA-seq identified two broad clusters of cells expressing a few house-keeping genes and many high variance genes (Supplementary Figure S7). Investigation of the cellular composition of RNA-Best-preserved cultured primary mouse pituitary cells showed co-expression of RNA markers classically associated with different pituitary cell types, thus supporting cell-to-cell heterogeneity within the pituitary and the existence of polyhormonal cells (Supplementary Figure S8). These results are consistent with previous co-detection of hormonal cell type markers in these cells using histological approaches (34,35). By assaying SC transcriptomes from RNA-Best-preserved samples of dissociated human brain biopsies using Drop-seq, we were similarly able to identify cell type composition in the neocortex from an epilepsy surgical resection specimen, as well as in the infiltrative neocortex from a glioblastoma surgical resection specimen (Supplementary Figure S9). Detection of rare B lymphocytes in the epileptic neocortex was confirmed by CD79a immunostaining. Overall, these results demonstrate the applicability of RNA-Best for SC transcriptome assays with diverse human and mouse primary tissues.

### Characterization of SC heterogeneity in IEG responses to GnRH using RNA-Best cell preservation

**Figure 4.**
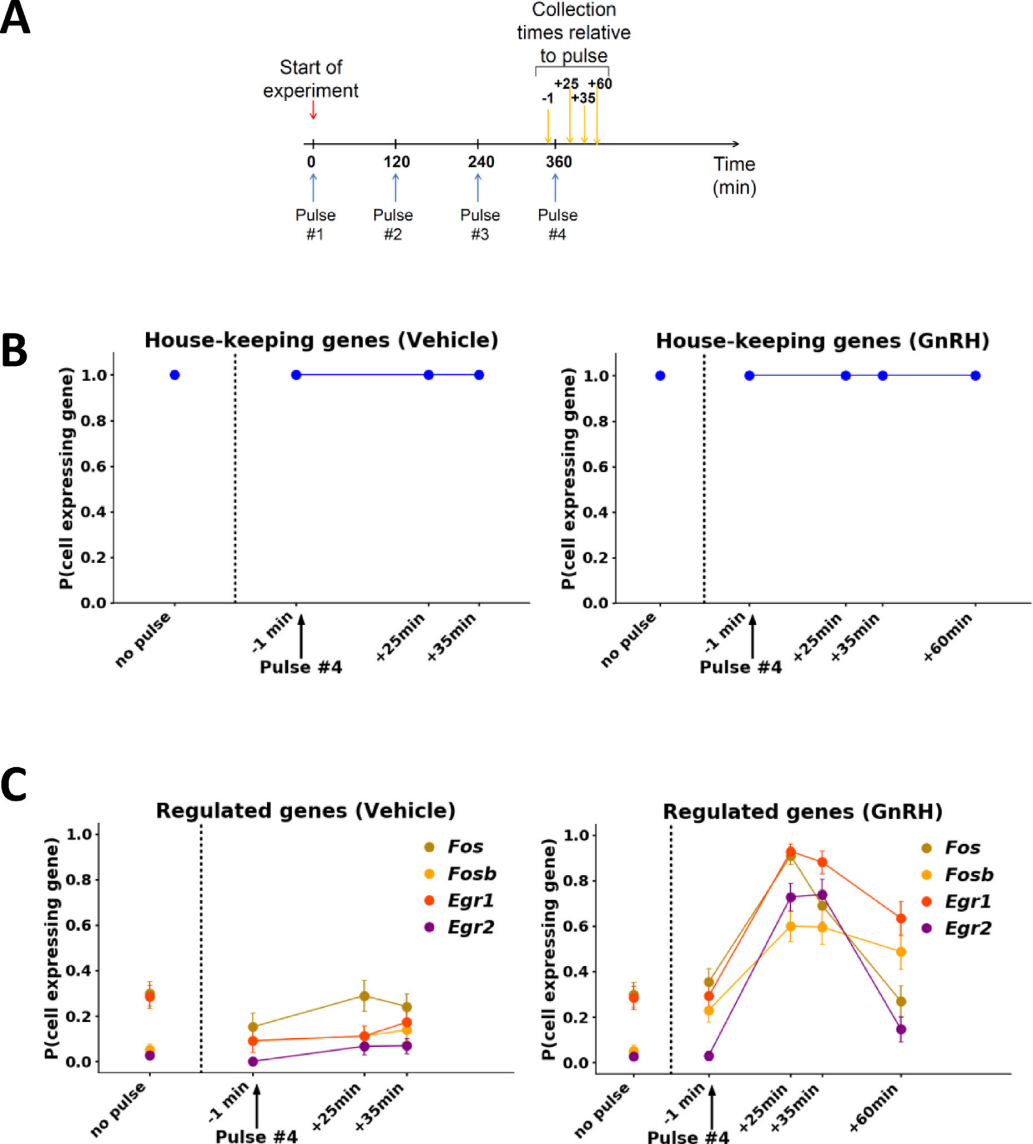
IEGs show bimodal SC responses to GnRH pulse stimulation. (**A**) Schematic of the time course experiment design. Cells were exposed to four pulses of GnRH (blue arrows) and collected at short time intervals around the 4th pulse (yellow arrows). (B, C) Graphs showing the probability for a SC to express a house-keeping gene (either *Eef1a, H2fz, Rps11*, or *Rps25*; (**B**)) or a regulated gene (as indicated; (**C**)) following either vehicle or GnRH treatment. Error bars are based on the binomial standard deviation on the number of gene-expressing cells. In C, *Right panel*, the +25 min and +35 min data points are statistically different from −1 min.

**Figure 5.**
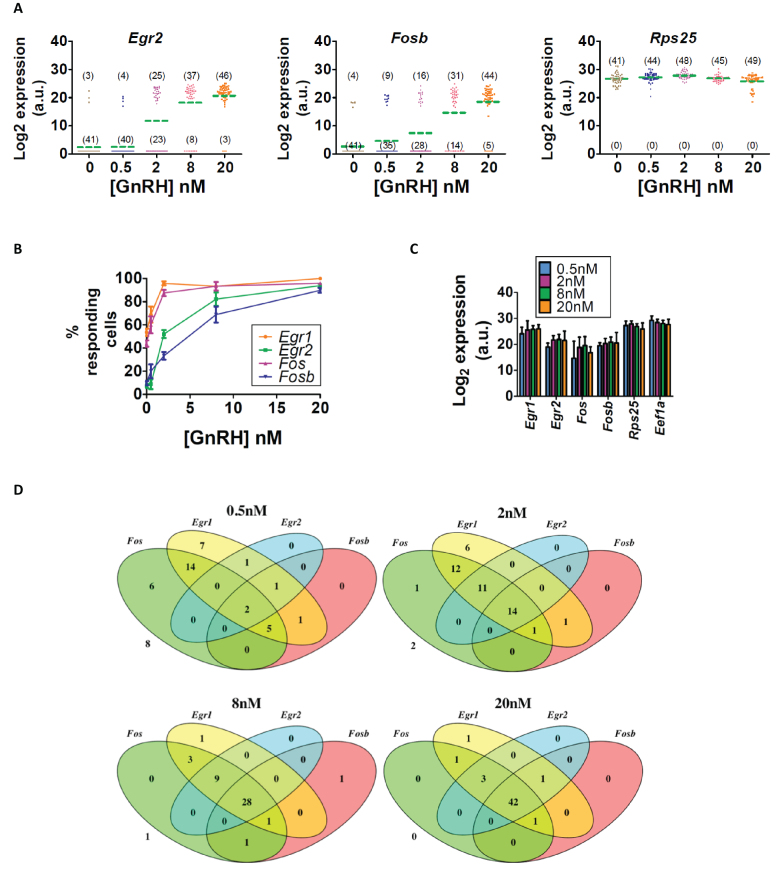
SC probability of IEG induction is concentration-dependent and varies for each IEG. (A–D) Cells were exposed to increasing concentrations of GnRH and collected 35 min after the fourth pulse. (**A**) Vertical scatter plots of *Egr2, Fosb*, and *Rps25* expression in SCs. In parentheses is indicated the number of gene-expressing (top) and non-gene expressing cells (bottom); each square represents a cell; the green dotted line signifies the average expression in all analyzed cells at a given GnRH concentration. (**B**) Plots of the percentages of cells expressing a regulated gene, as indicated. Error bars are based on the binomial standard deviation on the number of gene-expressing cells. (**C**) Bar graphs of average gene expression in gene expressing (i.e. induced) cells. Error bars represent standard deviation. ANOVA shows no significant differences. (**D**) Venn diagrams illustrating the overlap of *Fos, Egr1, Egr2*, and *Fosb* expression in all analyzed cells at +35 min.

The ability of RNA-Best to maintain SC transcriptome information allowed us to characterize SC heterogeneity in IEG responses to GnRH. In order to approximate the physiological pulsatile exposure paradigm, we exposed LβT2 cells to 5-min duration pulses of 2 nM GnRH every 2 h, as previously described (15), and collected samples at short time intervals around the fourth pulse (from −1 min to +60 min; Figure 4A). Two months later, over 400 SCs were IFC-isolated from these samples, and SC transcript levels of 54 genes were measured by IFC TaqMan assays (Supplementary Figures S10–S12, Supplementary Table S1). SC levels of the house-keeping genes showed similar unimodal distributions in control and GnRH-treated samples at all time points (Figure 4B, Supplementary Figure S11). In contrast, *Fos, Egr1*, and other regulated genes showed a bimodal distribution, with variable percentages of gene expressing vs. non-gene expressing cells (Figure 4C, Supplementary Figure S11). While non-gene expressing cells were predominant in control samples at all times, the proportion of gene expressing cells was dramatically higher at +25 min and +35 min compared to −1 min (Figure 4C). These results are consistent with our previous bulk temporal response data (15). To further delineate the gene regulatory mechanisms in SCs, we examined the effect of varying concentrations of GnRH (from 0.5 to 20 nM) on transcripts in samples collected at −1 min and +25 (or +35) min relative to the 4th pulse (Supplementary Figures S13–S16). Over 600 SCs were analyzed. All IEGs exhibited a concentration-dependent bimodal SC response pattern, with the proportion of gene expressing (or induced) cells increasing with increasing stimulus level (Figure 5A,B, Supplementary Figures S13 and S15). In contrast, all house-keeping genes showed comparable unimodal distributions at all concentrations (Figure 5A, Supplementary Figures S13 and S15). Notably, the level of transcript in cells showing induction of each gene was not increased with increasing concentration of GnRH (Figure 5C). Instead, the number of induced cells increased as concentration increased (Figure 5D, Supplementary Figure S15). Furthermore, we observed a hierarchical pattern of IEG induction, with *Egr1* and *Fos* being activated in SCs at lower concentrations than *Egr2* and *Fosb* (Figure 5B, D). Thus, these SC data resolve gene-specific all-or-none SC responses to increasing concentrations of GnRH exposed in a physiological pulsed pattern, and show that the SC probability of induction increases with concentration and differs for different GnRH-induced IEGs.

## DISCUSSION

The RNA-Best protocol greatly expands possibilities for both bulk and SC transcriptome studies by: (i) enabling analysis of samples in a clinical context, (ii) allowing for a geographical separation between the experimental and assay laboratories through easy storage and transportation, (iii) opening the prospects of complex time course experiments, which are needed for the study of highly regulated genes such as IEGs.

Importantly, RNA-Best allows, for the first time, the accurate SC measurement of IEG induction patterns. In our hands, while cell cryopreservation seemed to maintain RNA integrity, the thawing procedure appeared to result in increased IEG basal expression (see Figure 1C). The FRISCR method resulted in altered IEG expression profiles, suggesting that it may cause some significant RNA damage. FRISCR relies on cell fixation with paraformaldehyde, which generates covalent cross-links with proteins and nucleic acids (36,37). Formaldehyde is known to impair nucleic acid quality (38). Moreover, the reverse crosslinking step in FRISCR might result in relatively low percentages of protein-free RNA (39). Methanol fixation also resulted in altered IEG expression, despite the fact that alcohol-based non-crosslinking fixatives seem more effective at preserving nucleic acids (36). While we were preparing this manuscript, another cell preservation solution (Lomant's Reagent or DSP) was reported by others (22). Albeit promising, the use of DSP is associated with 3′ bias in sequencing coverage, suggesting that the cross-linker may cause some RNA damage.

While static/cyclic stretches or fluid stresses were previously shown to induce a transient increase in IEG transcript levels (17,18,40), we cannot completely rule out the possibility that trypsinization could represent a significant mechanical strain. However, it is worth noting that: (i) the same trypsinization procedure was applied to all samples in the time course experiment, (ii) only a small fraction of gonadotrope cells (<10%) exhibited IEG activation at the 0 and 0.5 nM GnRH concentrations, (iii) the vast majority of cells (>90%) were activated at the 20 nM concentration. Thus, while this background IEG activation might be caused by trypsinization, the stimulatory effect of GnRH is undeniable. Additionally, since trypsinization is carried out regardless of cell preservation, assessing its impact on IEG expression may be a challenging task and it is beyond the scope of this work.

Our results support a new model of immediate-early transcriptional responses to hormone stimulus in SCs. Our study demonstrates a switch-like induction of IEGs by GnRH, with the proportion of induced cells increasing with stimulus concentration and varying for different IEGs. To our knowledge, this is the first SC transcriptome study that consistently resolves the pattern of IEG induction responses to graded stimuli. Our findings represent an important contribution towards elucidating the quantitative and qualitative relationship between an inductive signal and the resulting transcriptional response.

Previous studies of IEG transcriptional responses were mostly population-based (8,9,12,13), thus masking cell-to-cell variation in gene expression. Later work using homologous gene recombination and live cell imaging to measure nascent RNA production in *Dictyostelium* cells supported an all-or-none response model where cells had different sensitivities to the inducer (4). In the present study, SC responses to pulsatile GnRH identify a digital activation model, yet the average level of expression in induced cells appears to be unaffected by increasing GnRH concentrations.

IEGs are activated by the MAPK signaling pathway following stimulation by an extracellular signal (1). A recent SC resolution study coupling optogenetic stimulation with live-cell reporters has highlighted the importance of the pulsatile ERK dynamics in IEG mRNA production, as recurrent pulses of ERK activity drive multiple cycles of IEG transcription, whereas continuous ERK activity results in lower transcription levels (41). These results and ours shed some light on how IEGs decode the pattern of ERK activity and varying concentrations of extracellular stimulus, respectively. Besides activating transcription factors, the MAPK signaling pathway also initiates chromatin/histone modifications that regulate IEG expression (for review, see (3)). For instance, *Fos* and *Egr1* induction in hippocampal neurons has been associated with increased histone phospho-acetylation without enhanced binding of CREB transcription factor at the promoters (42). As DNA methylation and histone modification are known to contribute to intercellular variability (for review, see (43)), a SC mapping of the epigenetic marks that are required for IEG activation would be needed. Additional factors that may influence IEG transcriptional responses in individual cells include transcription factor concentration, which may be stochastic, and cell type.

What are the physiological implications of an all-or-none, gene-specific hierarchical and probabilistic pattern of induction of IEGs? The periodicity of GnRH pulses varies throughout the female menstrual cycle, resulting in differential expression of the *Lhb* and *Fshb* genes (11). The aforementioned pattern of activation of IEGs likely contributes to the preferential induction of *Fshb* subunit gene by low frequency GnRH pulses (10,44). It is tempting to speculate that the gene-specific hierarchical pattern may be modified at high frequency pulses, thus favoring *Lhb* expression. Comparing the effect of high vs. low frequency GnRH pulses on IEG transcriptional responses might address this hypothesis. However, additional regulatory mechanisms may be involved, both at the level of IEG activation and downstream of IEG activation. Such mechanisms include but are not restricted to: selective epigenetic and/or microRNA-mediated silencing of IEGs, selective IEG protein degradation, selective inhibition of IEG proteins by repressors (for review, see (3)).

As the intercellular variation in membrane-localized GnRH receptor number is thought to be low in LβT2 cells (16), we speculate that the digital nature of cell response may stem from cell-to-cell variations in signaling effector concentrations, transcription factor concentrations, and/or in the epigenetic landscape of IEGs. Future studies combining SC transcriptomics, epigenomics and proteomics analysis will be needed to address these questions. To provide a more physiological context, similar studies should be extended to primary pituitary cells. To this end, we will test the applicability of RNA-Best for epigenomics and proteomics profiling. Finally, a recent study has revealed that single-nuclei preparation from a sub-region of the brain allows the detection of IEG expression associated with animal exposure to an environmental stimulus, whereas the whole-cell dissociation procedure elicits IEG expression independently of stimulus exposure (45).

The present study is limited by assay of 54 genes per cell in one cell type in response to graded concentrations of a single hormone. Further study of the SC dynamics of gene regulation using this more accurate technique and preservation protocol in other systems and for additional transcript classes is needed. In the present experiments, we find no evidence for regulation of the rate of transcription of IEGs at the level of the SC. Could it be possible that what has appeared to be dynamic gene regulation in population assays is entirely due to probabilistic quantal gene activation and suppression at the SC level? While this hypothesis is highly speculative based on the data presented in this study, it is a crucial question to now resolve in any attempt to elucidate the principles of the encoding and operation of the software programs of cells.

## DATA AVAILABILITY

The datasets (RNA-seq and SC RNA-seq data) generated during the current study are available in GEO (GSE111462).

## Supplementary Material

Supplementary Data
