# Supplementary material for: Single-cell stabilization method identifies gonadotrope transcriptional dynamics and pituitary cell type heterogeneity
Source: Nucleic Acids Res. 2018 Oct 24;46(21):11370–80. doi: 10.1093/nar/gky991 (PMC6265460; doi:10.1093/nar/gky991)
Supplement: Supplementary Data [file gky991_supplemental_files.zip › Suppl.Material_Text_Figs_09.07.18_NAR.pdf]

Single-cell stabilization method identifies gonadotrope transcriptional dynamics and pituitary cell type heterogeneity

Frederique Ruf-Zamojski, Yongchao Ge, Venugopalan Nair, Michel Zamojski, Hanna Pincas, Chirine Toufaily, Jessica Tome-Garcia, Marlon Stoeckius, William Stephenson, Gregory R. Smith, Daniel J. Bernard, Nadejda M. Tsankova, Boris M. Hartmann, Miguel Fribourg, Peter Smibert, Harold Swerdlow, Judith L. Turgeon, and Stuart C. Sealfon

Supplementary Material

1. Supplementary Methods

Imaging flow cytometry

SC images of fresh and RNA-Best stabilized LβT2 cells were acquired using an ISX imaging flow cytometer (Amnis, EMD Millipore). Cells were stained for DNA content with Hoechst 33342 (Invitrogen, Carlsbad CA, cat #3570; used at a 1:1000 dilution) at room temperature for 30 min, washed, resuspended in PBS before analysis. Cell size and morphology were assessed by forward and side scatter analysis and by quantifying bright field contrast. Images and data were collected for 10,000 events.

Human blood monocyte preparation

Monocyte-derived DC were obtained from buffy coats from healthy human blood donors following a standard protocol(1). Briefly, PBMC were isolated from buffy coats by Ficoll density gradient centrifugation, and CD14+ monocytes were immuno-magnetically purified.

Human cortex tissue collection

All human cortical tissue was obtained de-identified, from epilepsy or glioblastoma surgical resections, in accordance with the policies and regulations at the Icahn School of Medicine at Mount Sinai (ISMMS) and its institutional review board. The tissue was divided into several pieces, one of which was used for formalin fixation and another for fresh dissociation and RNA-Best stabilization. The fresh tissue was first dissociated into SC suspension by mechanical and enzymatic papain dissociation as previously described(2,3), and then stabilized in RNA-Best at 100,000 cells per ml.

Immunohistochemistry

Paraffin embedded sections (4 µm) fixed in 10% formalin were deparaffinized in xylene, with subsequent rehydration in decreasing gradient of ethanol. Primary antibody incubation with anti-CD79a (Leica CD79a Bond RTU, catalog #PA0192, 15-min incubation, heat-induced retrieval at high pH [Solution 2] for 20 min) and subsequent secondary antibody incubation and detection were performed using standard Leica Biosystems Bond^{TM} protocol and visualized on a confocal Nikon Eclipse Ci microscope.

Mini Drop-seq assay

SC Mini Drop-seq experiments were performed similarly as described(4). Ficoll PM-400 was added to the cell buffer to match fluid viscosity of the aqueous flows. Samples from dissociated human cortical tissue were processed as described in https://www.biorxiv.org/content/biorxiv/early/2017/05/22/140848.full.pdf. Libraries were sequenced on the Illumina HiSeq 2500 platform at the New York Genome Center. The SC RNA-seq data are deposited in GEO (GSE111462).

Mini Drop-seq data analysis

The initial data alignment and extracting UMI is done by the drop-seq tools from http://mccarrolllab.com/dropseq/. The t-SNE analysis (5,6) and the gene identification for annotating different clusters were performed using the implementation from Seurat package(4).

Primary mouse pituitary cells
Pituitaries were collected in Canada from randomly cycling pure C57BL/6 female mice aged 10-12 weeks. Animals were on a 12-hour on, 12-hour off light cycle, with lights starting at 7 AM and ending at 7 PM. Upon collection, pituitaries were digested with collagenase and dispersed with pancreatine. Cells were washed and plated into M199 medium supplemented with HEPES, 10% FBS, and antibiotics. About 500,000-700,000 cells were collected from 5 pituitaries, making up a sample. Samples were stored in 100 µl of RNA-Best and shipped to New York at 4°C for SC assays. Additionally, pituitaries were collected and acutely dissociated as described above, immediately stored in either RNA-Best or RNAlater, and shipped to New York for bulk RNA-seq assays. All mouse work at McGill University (Montreal, Quebec, Canada) was conducted under animal use protocol 5204, as approved by the Facility Animal Care Committee of the Goodman Cancer Research Centre.

Bulk RNA-seq assay
RNA was extracted from primary mouse pituitary cells stored in either RNA-Best (Sample 1) or RNAlater (Sample 2) using the Agilent Absolutely RNA kit. RNA quality was assessed on a Bioanalyzer. RNA-seq libraries were prepared using the Illumina Truseq LT mRNA kit (Illumina, #RS-122-2101). ERCCs (DNA Sequence Library for External RNA, Controls, #2374, National Institute of Standards and Technology) were added for analysis and quality assessment. Library quality control and quantification were assessed by spectrophotometry (Nanodrop), fluorometry (Qubit dsDNA High sensitivity Assay Kit), qPCR (Kapa Library Quantification Kit Illumina Platforms, Kapa Biosystems, #KK4835), and on a Bioanalyzer (High-Sensitivity DNA Bioanalyzer kit, Agilent). Additionally, the quality of each library was assessed by qPCR of selected genes. A total of 2 libraries (one from Sample 1, the other from Sample 2) were pooled at equal concentrations, and the pooled sample (20 µl, 10 nM) was sequenced at the Epigenomics Core of Weill Cornell Medical College on Illumina HiSeq 2500 v3 using 51 bp single reads. The RNA-seq data are deposited in GEO (GSE111462).

Fluidigm C1 for SC RNA-seq
Primary mouse pituitary cells were stabilized in RNA-Best at 400,000 cells/ml, filtered through a 10-µm filter, and prepared for assay on the C1 according to the standard Fluidigm protocol for the SMART-Seq V4 Ultra low Input RNA Kit (Clontech, 032416), using a small 5-10 µm primed chip. A 3:2 ratio of RNA-Best:C1 suspension reagent was prepared. Amplified cDNAs from individual cells were harvested into 10 µl of C1 DNA dilution reagent from Fluidigm. SC libraries were prepared using the Nextera RT DNA library kits (Illumina). Libraries were individually checked for quality on a Bioanalyzer before being pooled for sequencing. The SC RNA-seq data are deposited in GEO (GSE111462).

2. Supplementary Figures

Supplementary Figures S1-S16 (displayed further below)

3. Supplementary Table

Supplementary Table 1: List of qPCR primers used in the microfluidic system (see attached Excel file)

References

1. Borderia, A.V., Hartmann, B.M., Fernandez-Sesma, A., Moran, T.M. and Sealfon, S.C. (2008) Antiviral-activated dendritic cells: a paracrine-induced response state. *J Immunol*, **181**, 6872-6881.
2. Tome-Garcia, J., Doetsch, F. and Tsankova, N.M. (2017) FACS-based Isolation of Neural and Glioma Stem Cell Populations from Fresh Human Tissues Utilizing EGF Ligand. *Bio Protoc*, **7**.
3. Tome-Garcia, J., Tejero, R., Nudelman, G., Yong, R.L., Sebra, R., Wang, H., Fowkes, M., Magid, M., Walsh, M., Silva-Vargas, V. *et al.* (2017) Prospective Isolation and Comparison of Human Germinal Matrix and Glioblastoma EGFR(+) Populations with Stem Cell Properties. *Stem Cell Reports*, **8**, 1421-1429.
4. Macosko, E.Z., Basu, A., Satija, R., Nemesh, J., Shekhar, K., Goldman, M., Tirosh, I., Bialas, A.R., Kamitaki, N., Martersteck, E.M. *et al.* (2015) Highly Parallel Genome-wide Expression Profiling of Individual Cells Using Nanoliter Droplets. *Cell*, **161**, 1202-1214.
5. Amir el, A.D., Davis, K.L., Tadmor, M.D., Simonds, E.F., Levine, J.H., Bendall, S.C., Shenfeld, D.K., Krishnaswamy, S., Nolan, G.P. and Pe'er, D. (2013) viSNE enables visualization of high dimensional single-cell data and reveals phenotypic heterogeneity of leukemia. *Nature biotechnology*, **31**, 545-552.
6. van der Maaten, L. and Hinton, G. (2008) Visualizing Data using t-SNE. *Journal of Machine Learning Research*, **9**, 2579-2605.

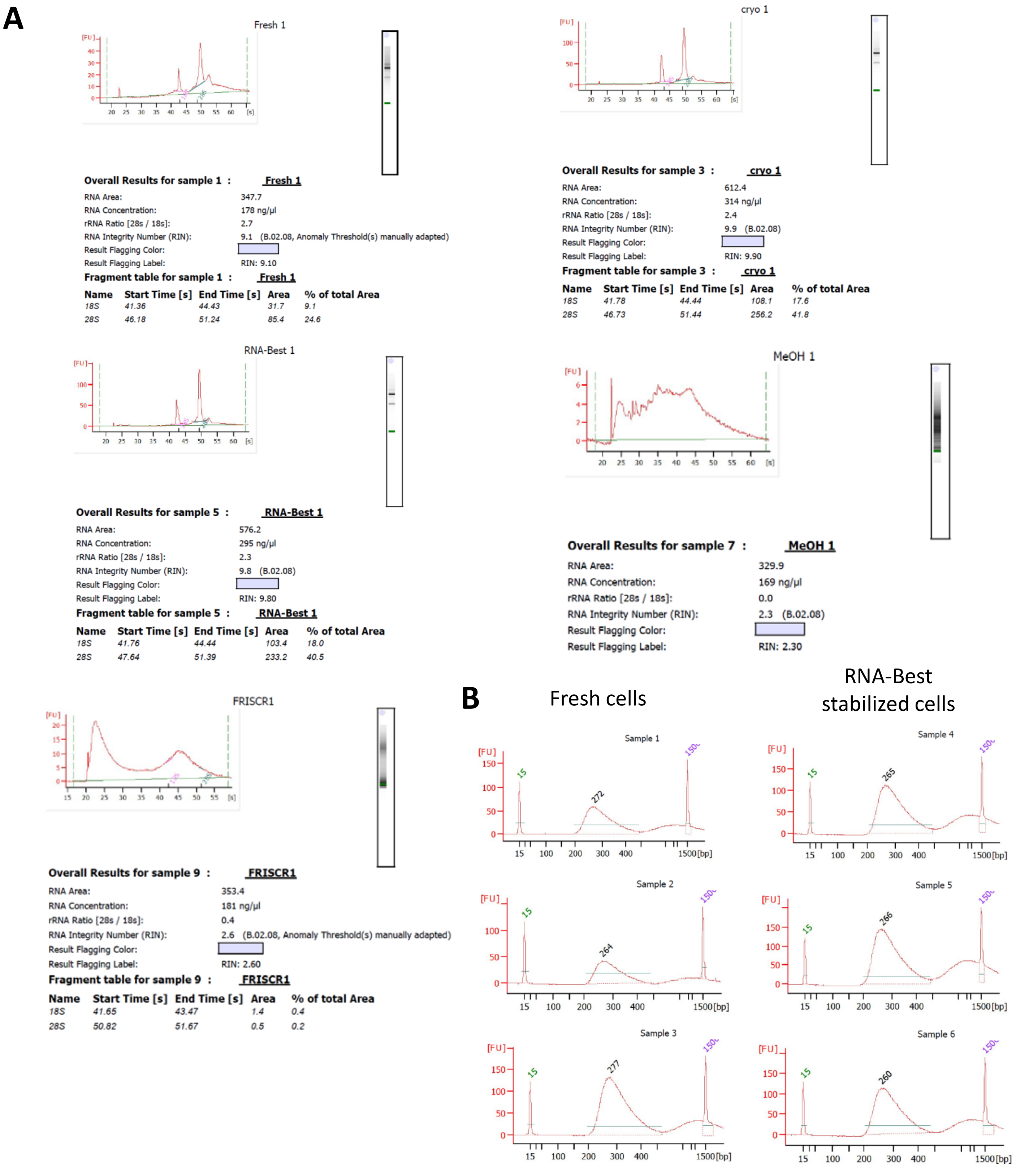

Supplementary Figure S1: Quality assessment of RNA and RNA-seq libraries following RNA-Best cell stabilization

(**A**) Comparison of Bioanalyzer traces of RNA extracted from fresh cells, RNA-Best-preserved cells, and cells preserved using other protocols (cryopreservation [cryo], methanol fixation [MeOH], and FRISCR). The indicators of RNA integrity (28S:18S ribosomal RNA ratio and RIN) and quantity (RNA concentration) are provided. (**B**) Comparison of Bioanalyzer traces of RNA-seq libraries prepared from 3 fresh vs. 3 RNA-Best-preserved cell samples.

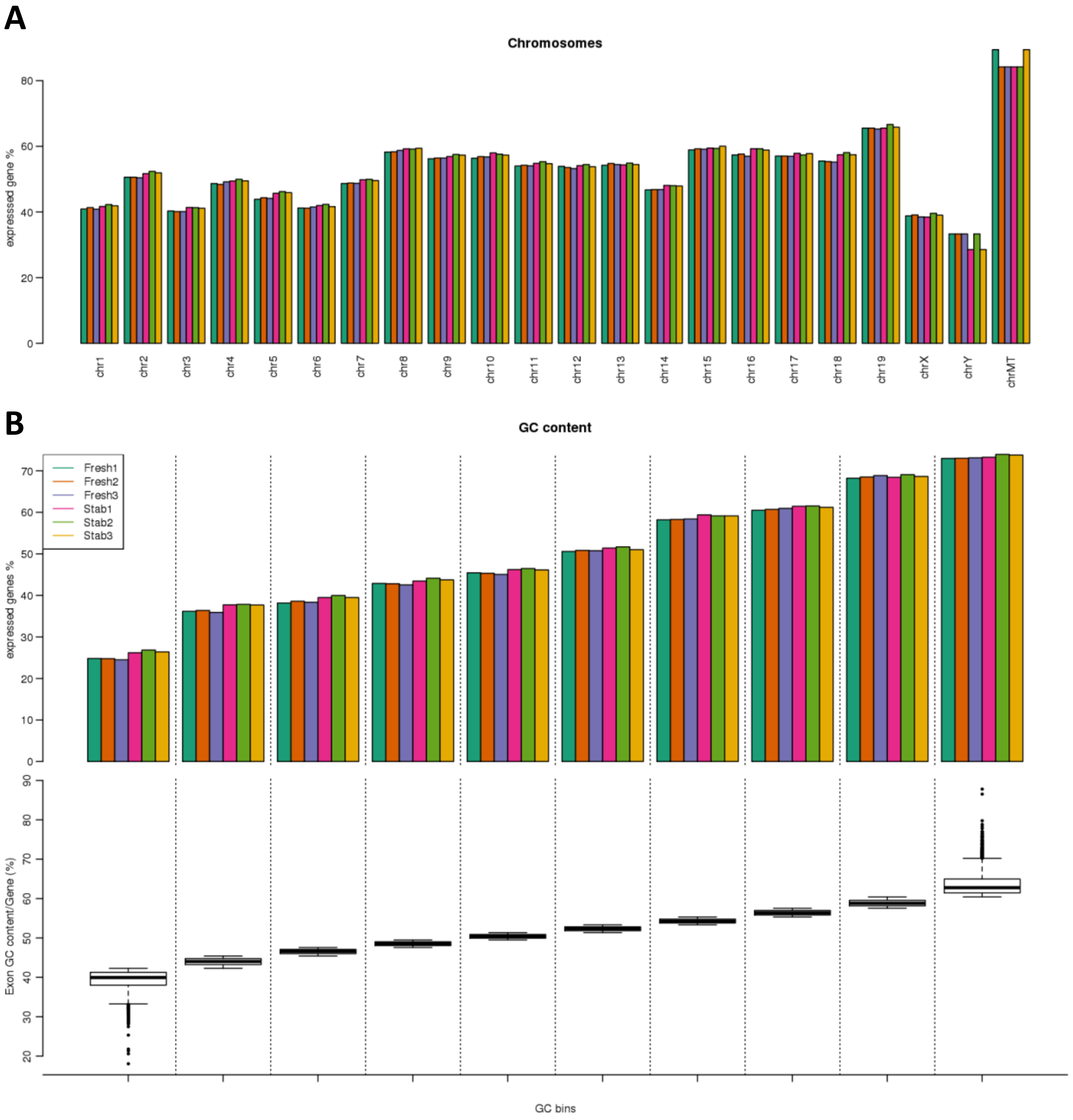

Supplementary Figure S2: Chromosomal transcript representation bias and GC coverage in bulk RNA-seq samples from LβT2 gonadotropes stabilized in RNA-Best

Comparison of chromosomal transcript representation bias (**A**) and GC coverage (**B**) in 3 RNA-Best- vs. 3 fresh samples following GnRH treatment.

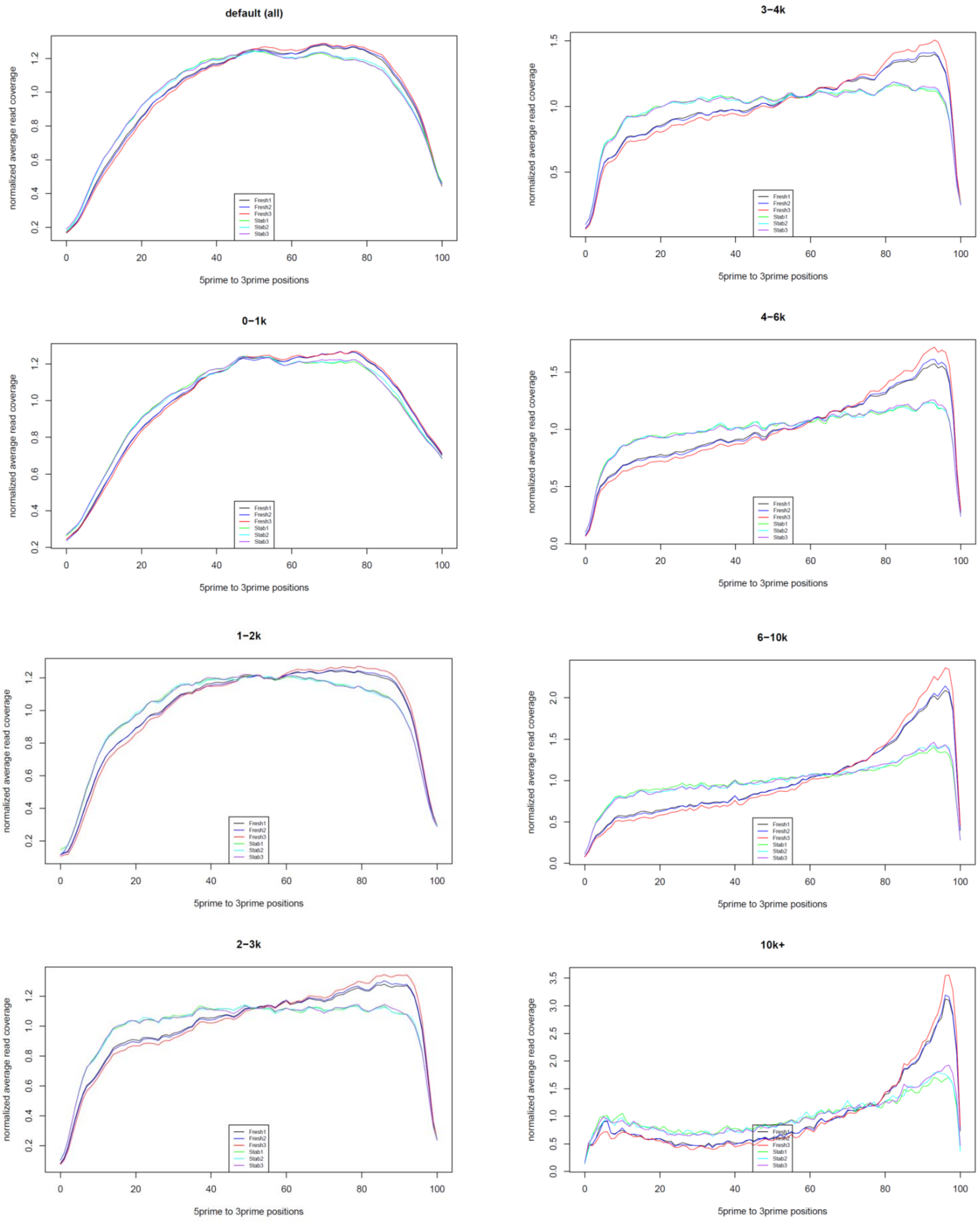

Supplementary Figure S3: Read coverage across all predicted transcript lengths in bulk RNA-seq samples from LβT2 gonadotropes stabilized in RNA-Best

Comparison of reads across predicted transcript lengths ranging from 0 to 10k^{+} in 3 RNA-Best- vs. 3 fresh samples following GnRH treatment.

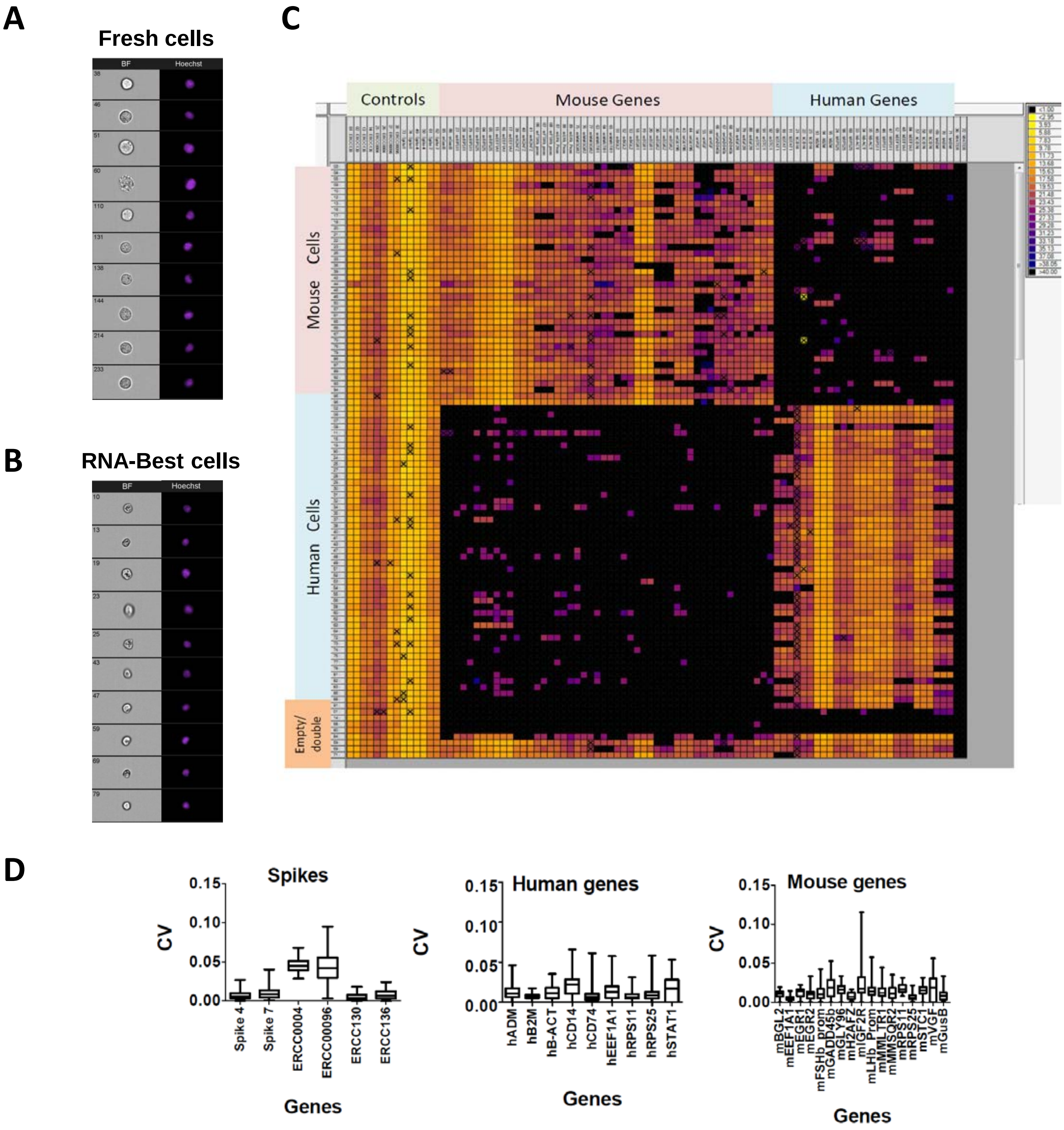

Supplementary Figure S4: Quality assessment of SCs and SC gene expression data following RNA-Best cell stabilization

(**A**,**B**) Imaging flow cytometry analysis of fresh (**A**) and RNA-Best-stabilized LβT2 cells (**B**). Cell size and morphology were evaluated by side scatter (SSC) analysis and by quantifying bright field (BF) contrast. Cells were stained with Hoechst to label nuclei. (**C**,**D**) Quality evaluation of SC qPCR measurements obtained using the Fluidigm IFC C1 system. (**C**) Heat map of External RNA *Controls* Consortium (ERCC) RNA spike-in controls, mouse and human genes expressed in a 50:50 mixture of human blood monocytes and murine LβT2 cells. (**D**) Analysis of the coefficient of variation (CV) for average Ct values of SC qPCR replicates of ERCCs (Spikes), human-specific transcripts (Human genes), and mouse-specific transcripts (Mouse genes).

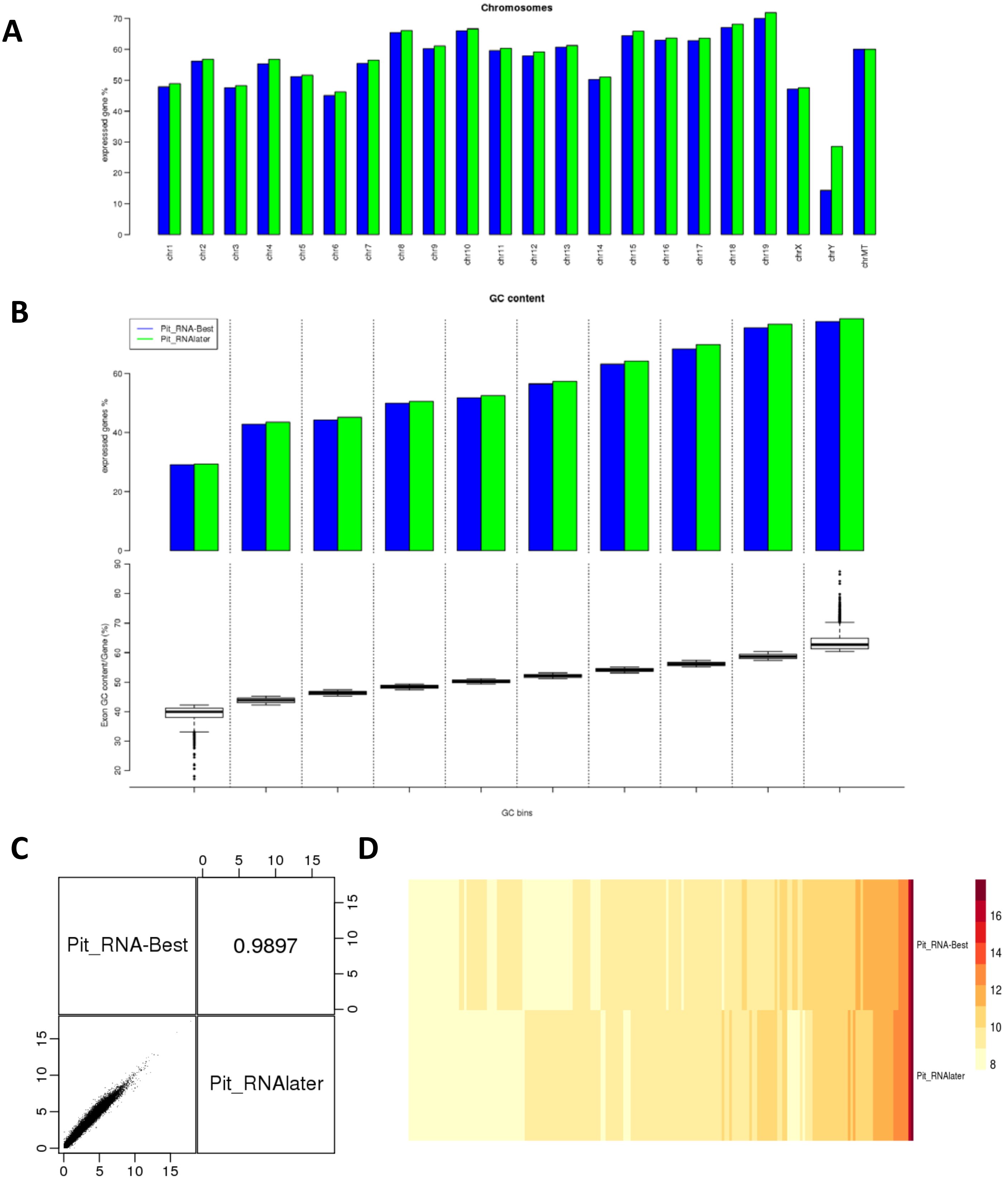

Supplementary Figure S5: Quality control data of bulk RNA-seq samples from dissociated mouse pituitaries stabilized in RNA-Best

(**A,B**) Comparison of chromosomal transcript representation bias (**A**) and GC content distribution (**B**) in dissociated mouse pituitaries stabilized in RNA-Best vs. RNAlater, an established RNA-stabilizing reagent. (**C**) Pairwise correlation between gene expression data from the same samples as in **A**. (**D**) Heat map of highly expressed genes found in the same samples as in **A**.

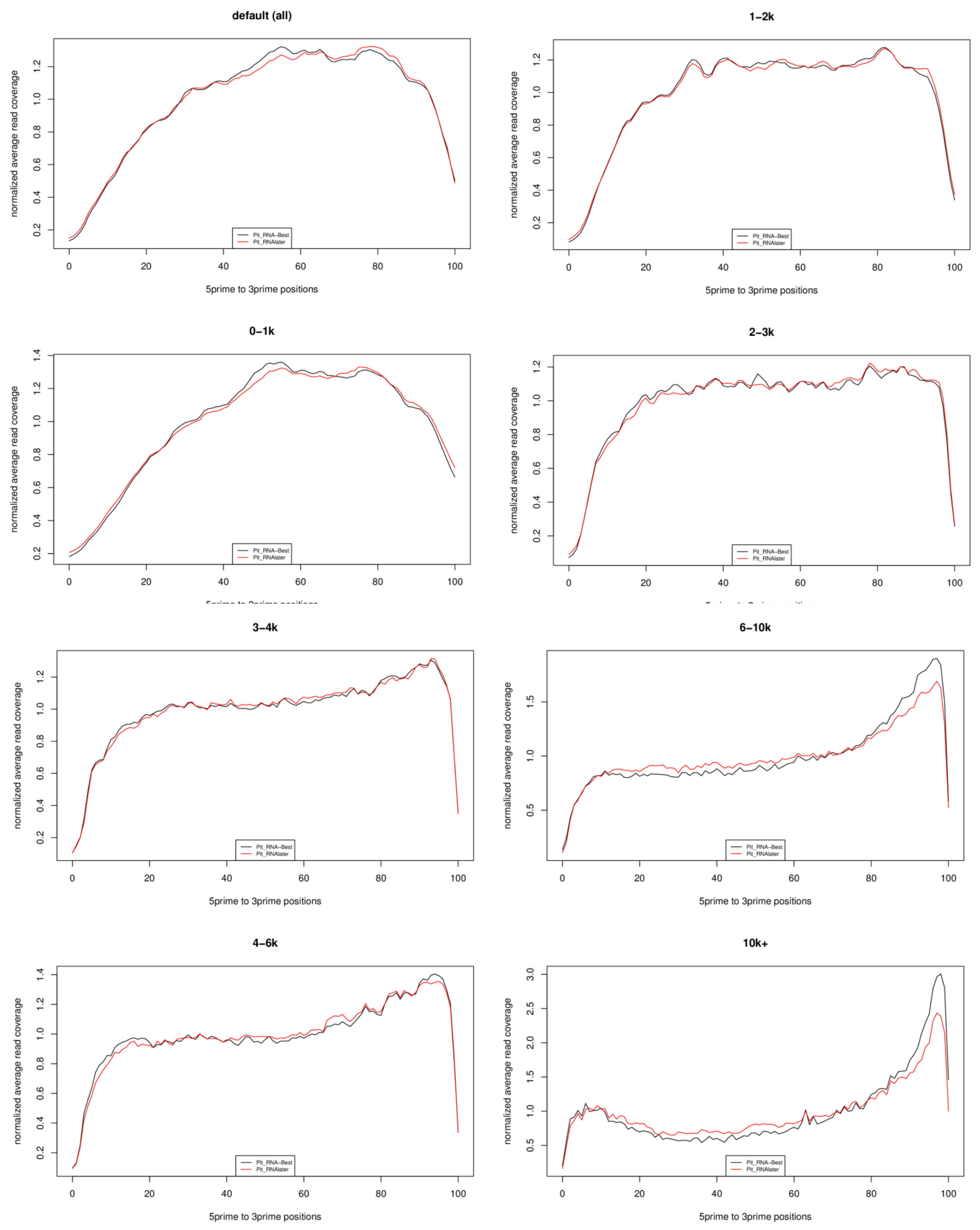

Supplementary Figure S6: Read coverage across all predicted transcript lengths in bulk RNA-seq samples from dissociated pituitaries stabilized in RNA-Best

Comparison of reads across predicted transcript lengths ranging from 0 to 10k$^+$ in dissociated mouse pituitaries stabilized in RNA-Best vs. RNAlater.

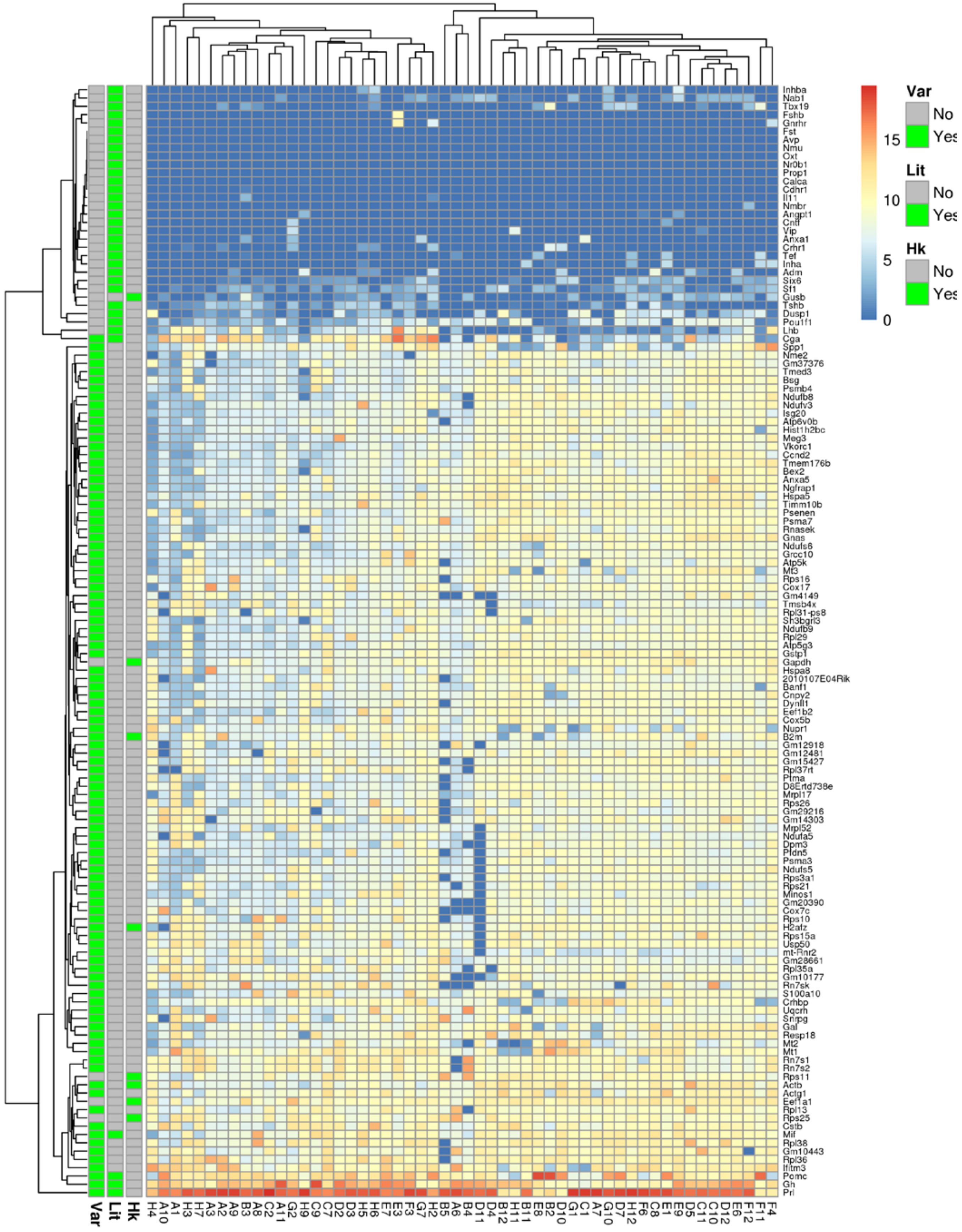

Supplementary Figure S7: Analysis of SC heterogeneity in primary mouse pituitary cultures stabilized in RNA-Best

Heat map of house-keeping genes and high variance genes found in primary mouse pituitary cell cultures following IFC-SC isolation and SC RNA-seq.

A

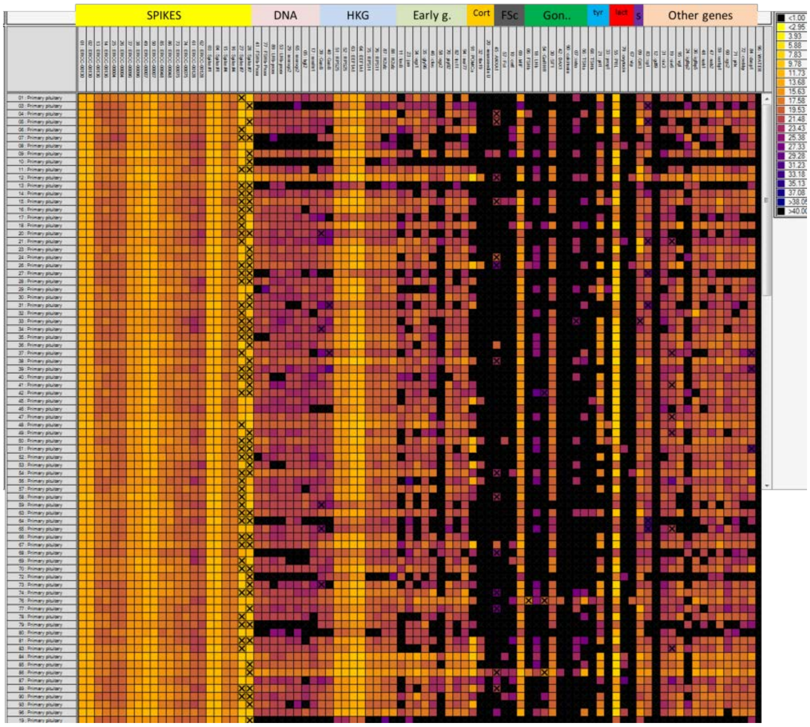

B

Cell	*Gnrhr*	*Lhb*	*Fshb*	*Cga*	*Prl*	*Gh*	*Tshb*	*Pomc*
1	15.3	1.0	17.5	21.5	32.7	22.7	1.0	20.8
2	13.9	11.4	1.0	18.6	31.3	19.9	1.0	18.2
3	22.2	16.5	24.7	28.4	24.5	21.8	17.1	21.9
4	24.9	18.6	24.0	30.8	33.1	24.8	1.0	29.4
5	16.5	1.0	1.0	19.8	31.5	20.5	1.0	17.3
6	19.4	17.8	1.0	26.3	33.8	24.6	1.0	19.3
7	1.0	1.0	19.5	22.2	32.3	22.8	1.0	17.0
8	1.0	14.7	17.5	24.3	32.5	22.1	1.0	28.7
9	1.0	15.0	1.0	19.4	32.1	19.9	18.8	1.0
10	1.0	1.0	1.0	17.9	31.5	20.7	17.5	28.2
11	1.0	15.0	1.0	21.2	31.4	26.4	17.5	1.0

Log_2 expression

High

Low

Cell	*Gnrhr*	*Lhb*	*Fshb*	*Cga*	*Prl*	*Gh*	*Tshb*	*Pomc*
1	10.6	16.0	10.1	13.9	16.6	15.9	4.5	13.3
2	5.7	11.5	0.0	2.4	11.3	12.8	0.0	4.9
3	6.6	9.1	0.0	16.5	15.4	10.1	3.4	15.6
4	0.0	3.8	0.0	14.4	16.9	14.9	0.0	15.8
5	0.0	5.1	0.0	6.2	18.7	10.3	4.1	12.8
6	0.0	9.2	0.0	13.1	18.2	13.4	5.0	13.3
7	0.0	10.5	0.0	13.1	18.9	14.2	3.6	13.0
8	0.0	7.0	0.0	10.1	17.3	16.8	2.4	13.2
9	0.0	7.2	0.0	15.5	18.1	15.0	5.2	14.4
10	0.0	11.1	0.0	14.9	18.5	10.2	1.3	11.0

$Log_2(TPM+1)$

High

Low

Supplementary Figure S8: Analysis of cellular composition in primary mouse pituitary cultures stabilized in RNA-Best

(**A**) Heat map of selected genes analyzed in primary mouse pituitary cultures treated with 5 nM GnRH following IFC-SC isolation and SC qPCR using a panel for pituitary gene markers. (**B**) Tables displaying gonadotrope-specific markers that are co-expressed in selected pituitary SCs. All cells expressing either *Gnrhr*, *Fshb*, or *Tshb* were selected for display. Both nominal cell copy numbers (*Top*) from SC qPCR and average transcripts per million from SC RNA-seq (*Bottom*) are shown.

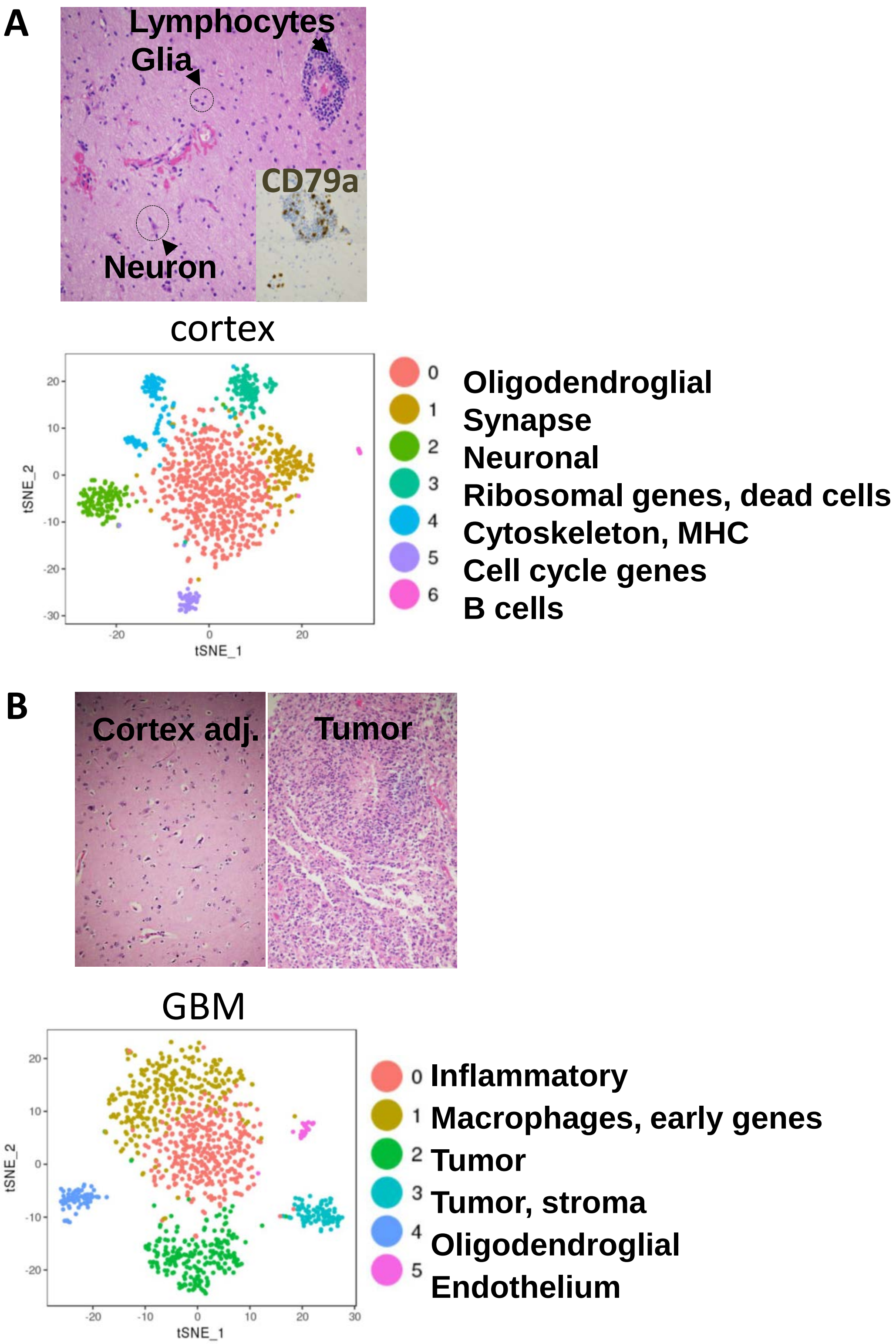

Supplementary Figure S9: Analysis of SC heterogeneity in dissociated human brain tissue stabilized in RNA-Best

(**A**) *Top*, Micrograph of a cerebral cortical tissue section, with anti-CD79a antibody staining (*inset*). *Bottom*, Clustering of cells from the dissociated cortical tissue into distinct cell populations, rendered on a two-dimensional tSNE representation based on a clustering analysis of Mini Drop-seq SC RNA-sequencing data. Colors indicate cell type or gene group. (**B**) *Top*, Micrograph of tissue sections of a glioblastoma multiforme (GBM, Tumor) and its adjacent cortex (Cortex adj.). *Bottom*, Clustering of cells from the dissociated GBM tissue into distinct cell populations on a tSNE plot based on SC RNA-sequencing.

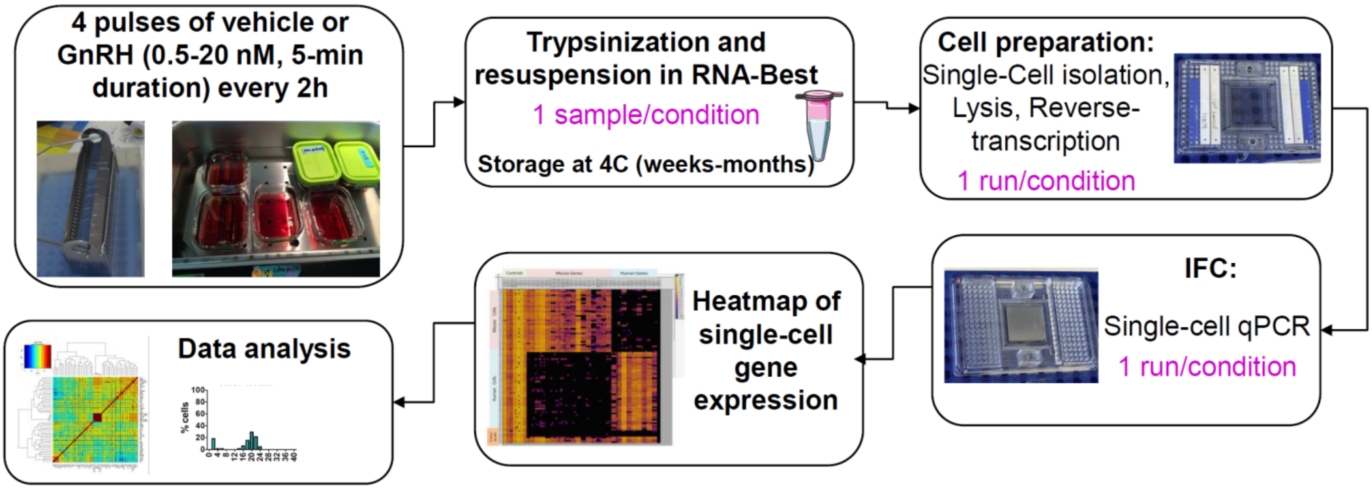

Supplementary Figure S10: Schematic of the experimental workflow used for characterizing SC heterogeneity in IEG responses to GnRH pulse stimulation

The experimental workflow comprises: i) time course experiments where cells were exposed to 4 pulses of GnRH (2 nM, 5-min duration) every 2 h and collected at short time intervals around the 4th pulse, ii) stabilization of the cells in RNA-Best and storage of stabilized samples at 4°C, iii) use of an integrated fluidic circuit (IFC) C1 system for SC isolation and high-throughput SC qPCR up to two months later. Heat maps of SC gene expression data are generated, and further data analysis is performed.

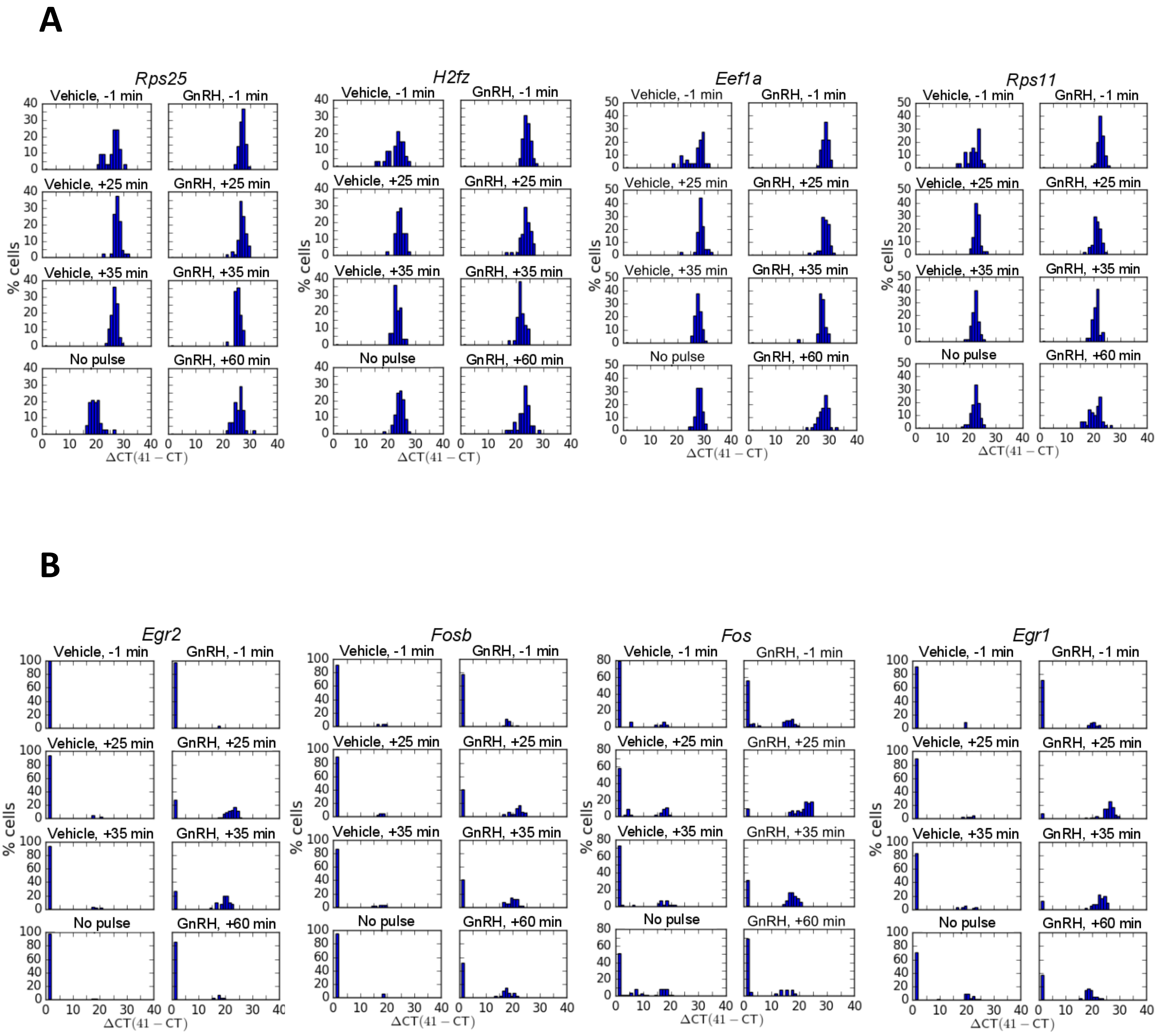

Supplementary Figure S11: Characterization of SC heterogeneity in the response of IEGs to GnRH pulse stimulation

This is the same experiment as the one illustrated in **Fig. 4B,C**. (**A**,**B**) Histograms of the transcript levels of house-keeping genes (**A**) and IEGs (**B**) in all analyzed SCs following either vehicle, no pulse, or GnRH treatment. Cells were exposed to 4 pulses of GnRH (2 nM, 5-min duration) every 2 h and collected at short time intervals around the fourth pulse (from -1 min to +60 min), as depicted in **Fig. 4A**.

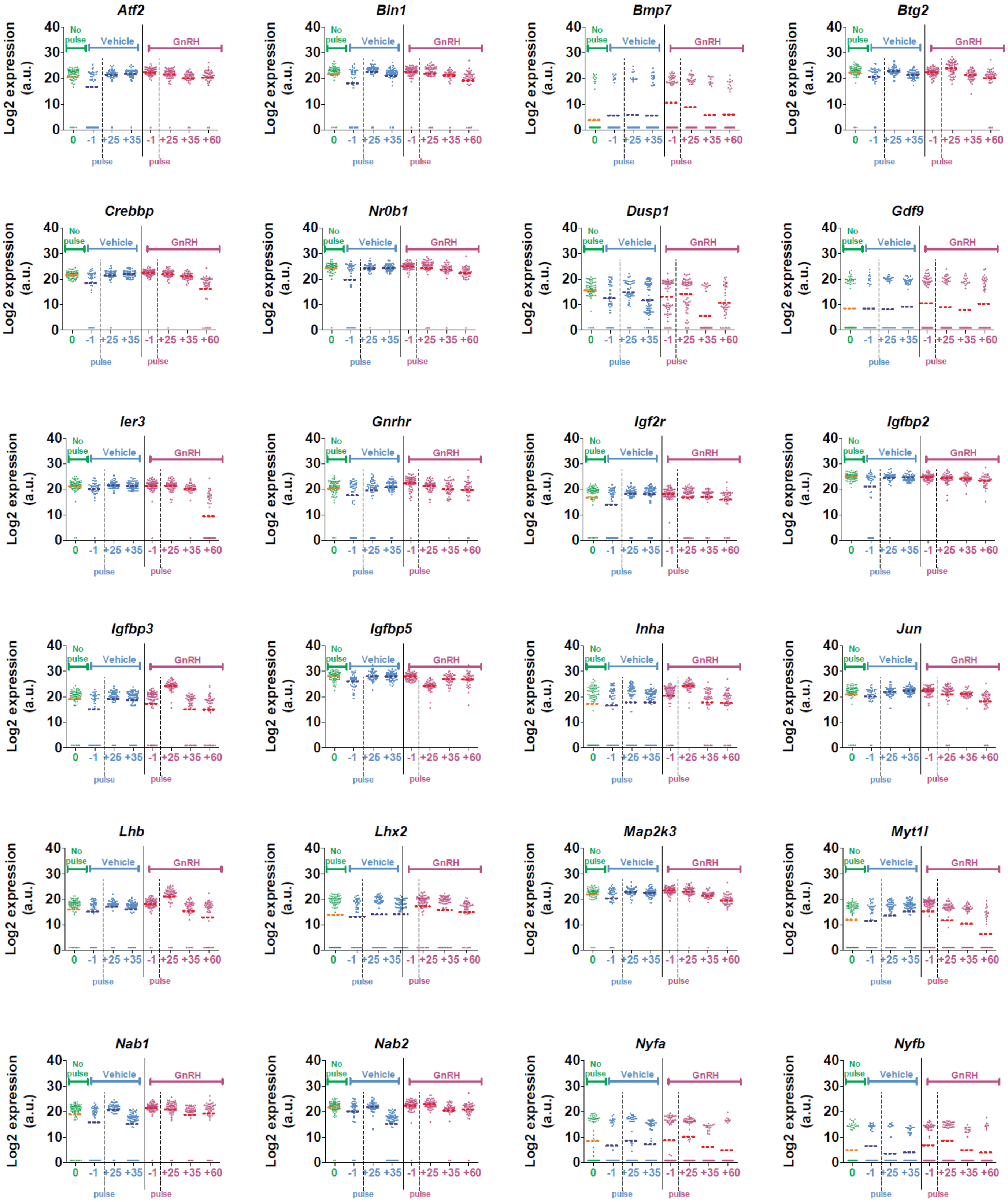

Supplementary Figure S12: Analysis of the SC response of 45 genes to GnRH pulse stimulation (Part 1)

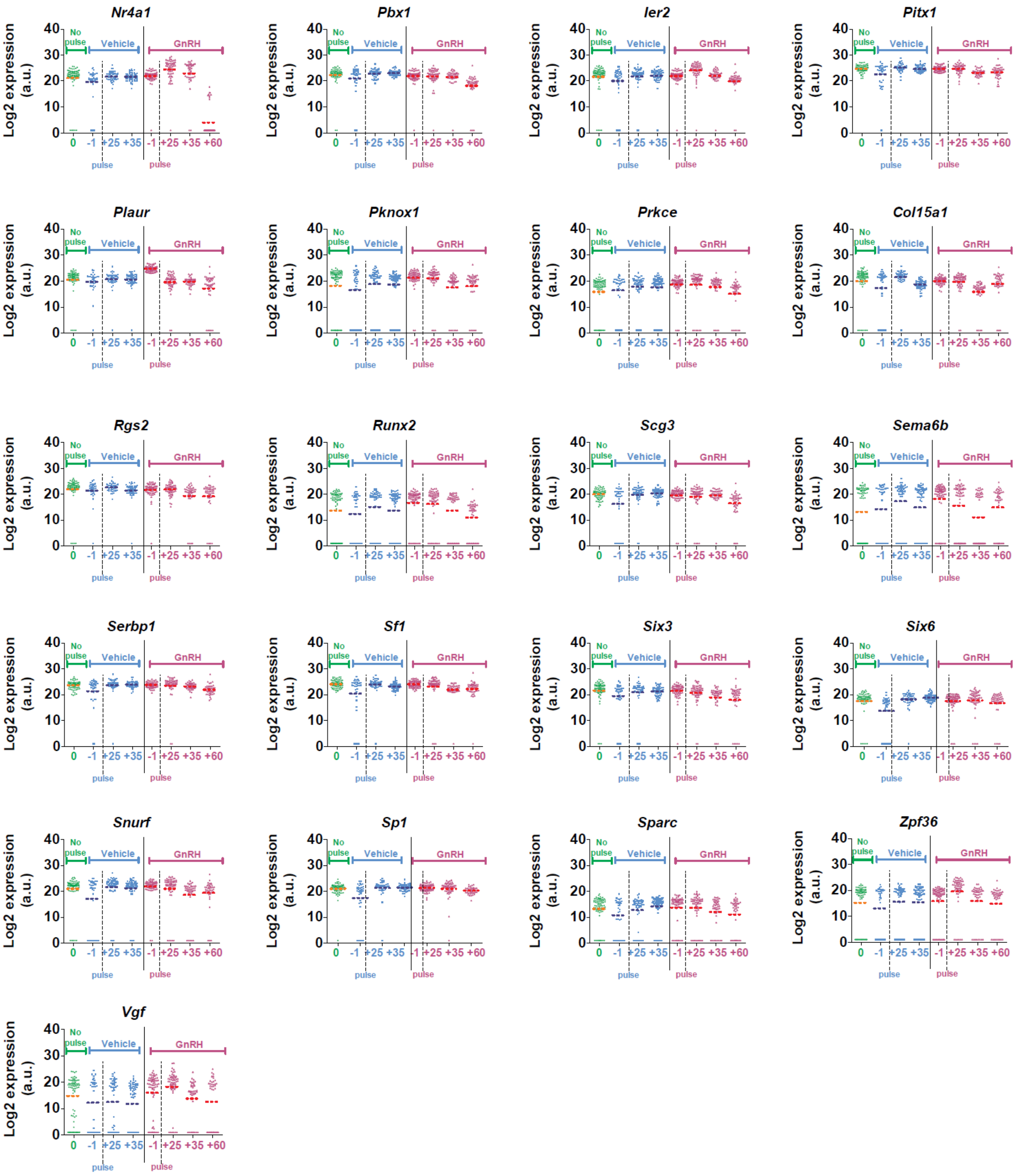

Supplementary Figure S12: Analysis of the SC response of 45 genes to GnRH pulse stimulation (Part 2)

Vertical scatter plots of gene expression in SCs following either no pulse, vehicle or GnRH treatment are shown for 45 genes. Cells were exposed to 4 pulses of GnRH (2 nM, 5-min duration) every 2 h and collected at short time intervals around the fourth pulse (from -1 min to +60 min), as depicted in **Fig. 4A**. The dotted line signifies average gene expression in all analyzed cells under each experimental condition. Data are from the same experiment as the one presented in **Fig. 4B,C**.

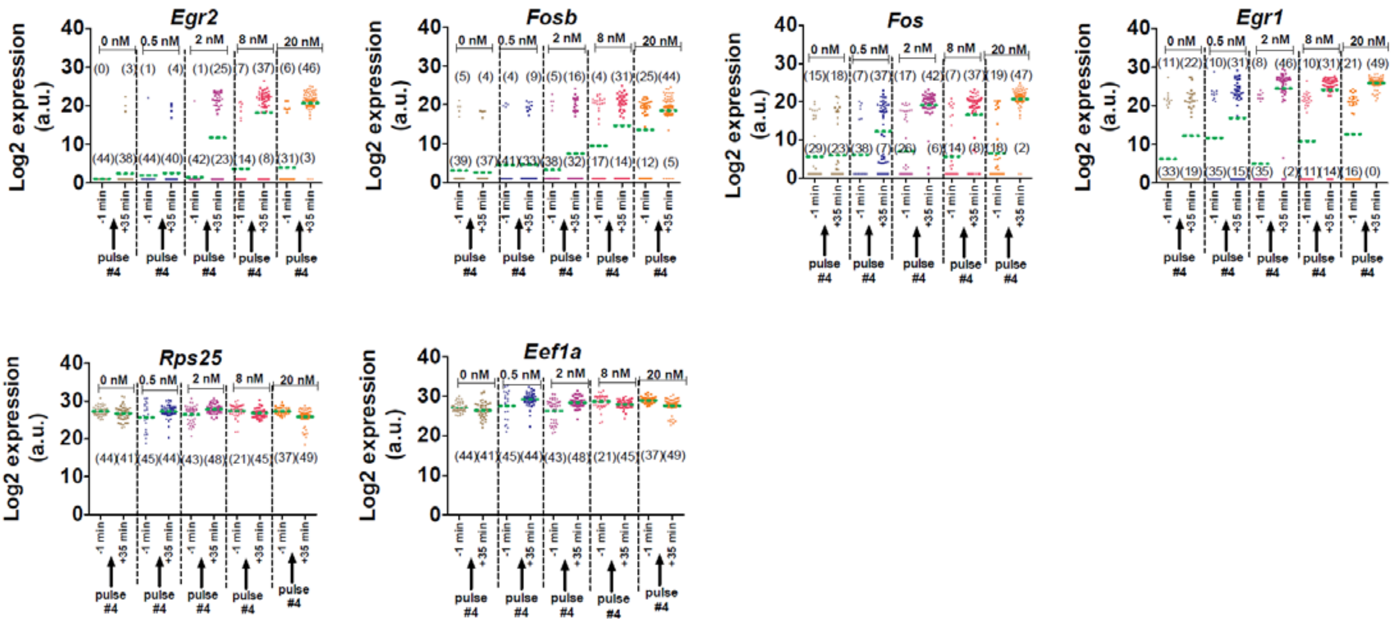

Supplementary Figure S13: Characterization of SC heterogeneity in the response of IEGs to different concentrations of GnRH

Vertical scatter plots of IEG (*Top*) and housekeeping gene expression (*Bottom*) in SCs following exposure to increasing concentrations of GnRH and collection at either -1 min or +35 min relative to pulse #4. A minimum of 40 cells were analyzed per condition. In parentheses is indicated the number of gene-expressing (top) and non-gene expressing cells (bottom). Each cell is represented either by a dot (-1 min) or a square (+35 min); the green dotted line signifies average gene expression in all analyzed cells under each experimental condition. Data are from the same experiment as the one presented in **Fig. 5A**.

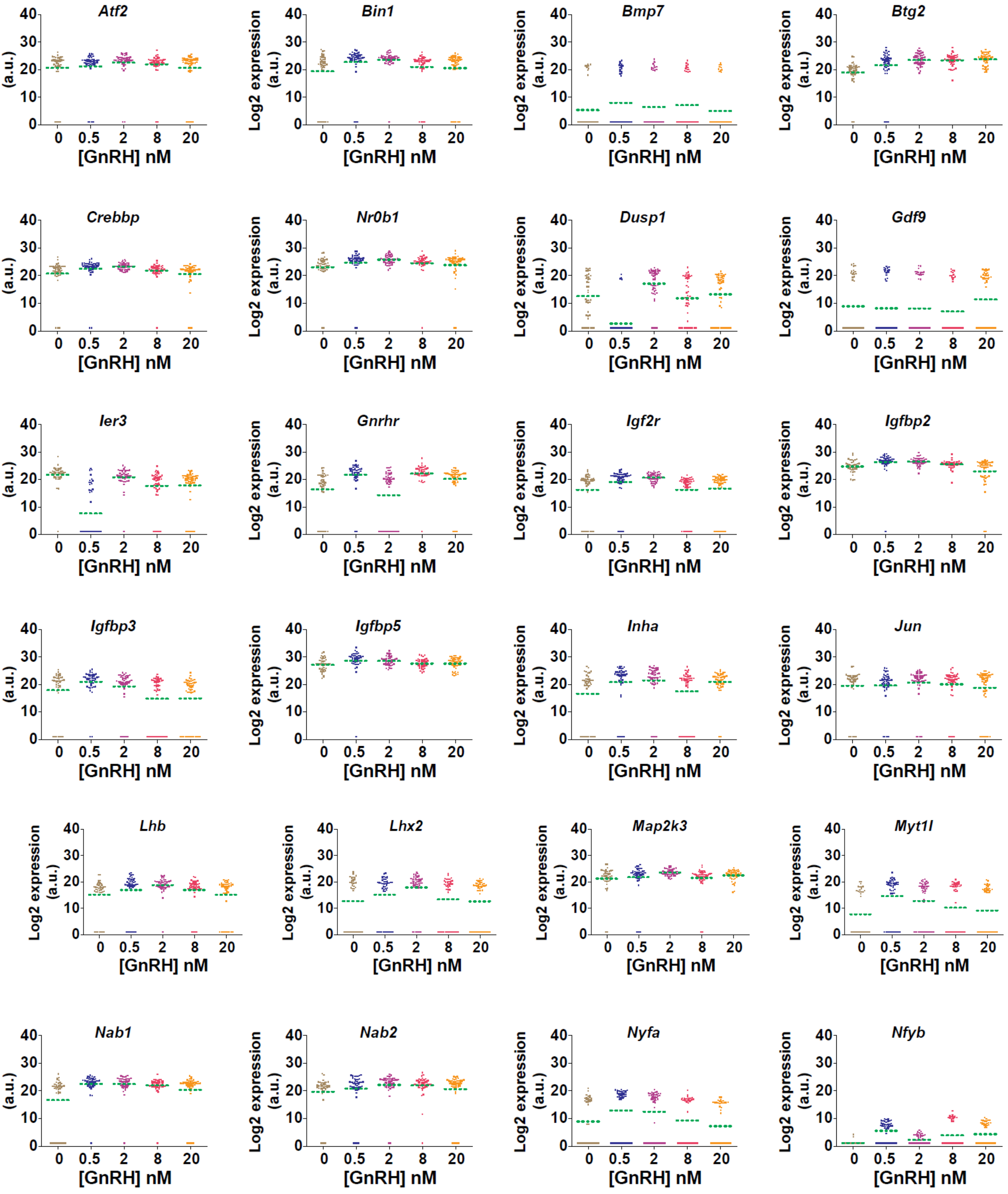

Supplementary Figure S14: Analysis of the SC response of 45 genes to different concentrations of GnRH (Part 1)

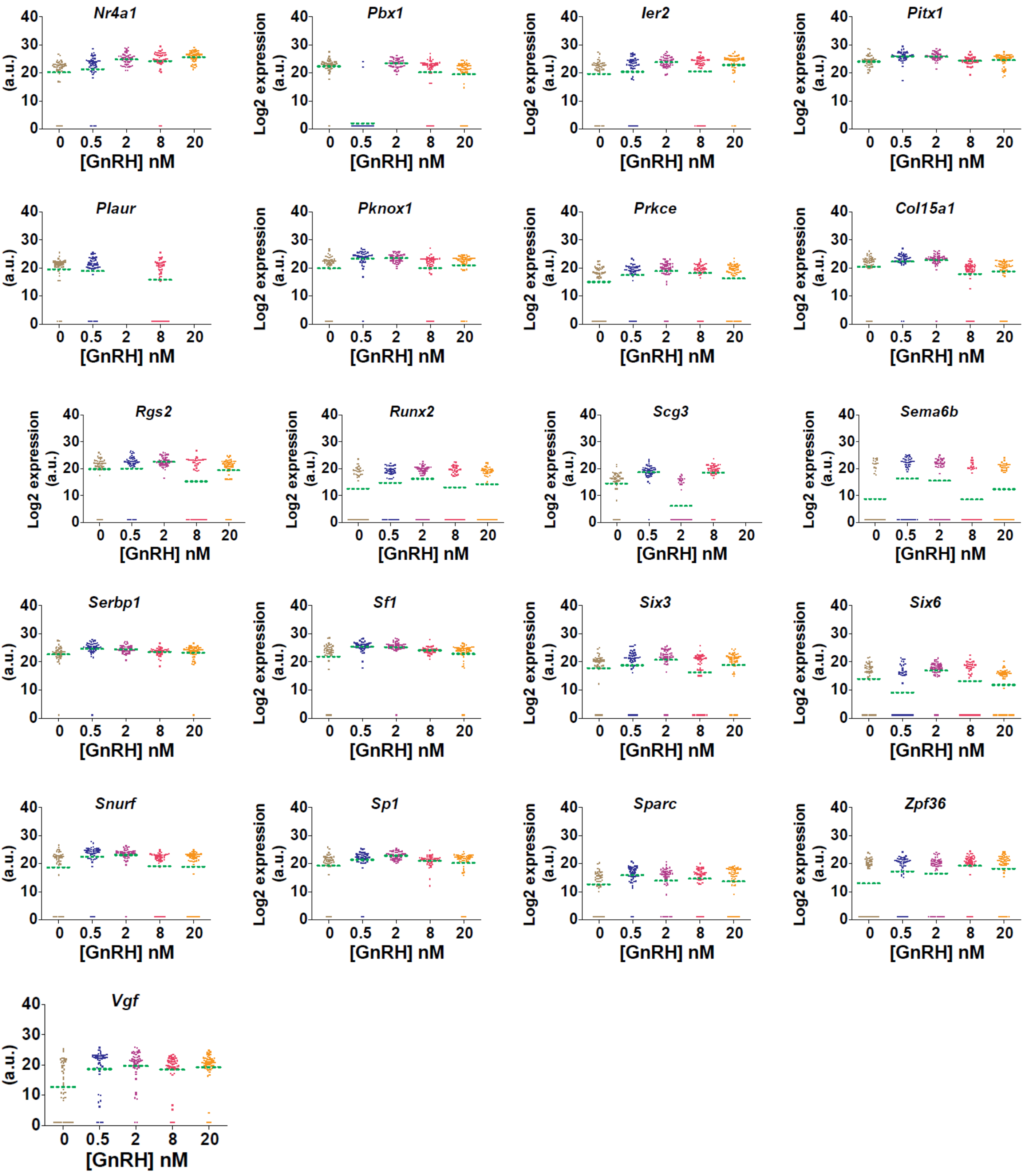

Supplementary Figure S14: Analysis of the SC response of 45 genes to different concentrations of GnRH (Part 2)

Vertical scatter plots of gene expression in SCs following exposure to increasing concentrations of GnRH and collection at +35 min relative to pulse #4 are shown for 45 genes. A minimum of 40 cells were analyzed per condition. The green dotted line signifies average gene expression in all analyzed cells under each experimental condition. Data are from the same experiment as the one presented in **Fig. 5A**.

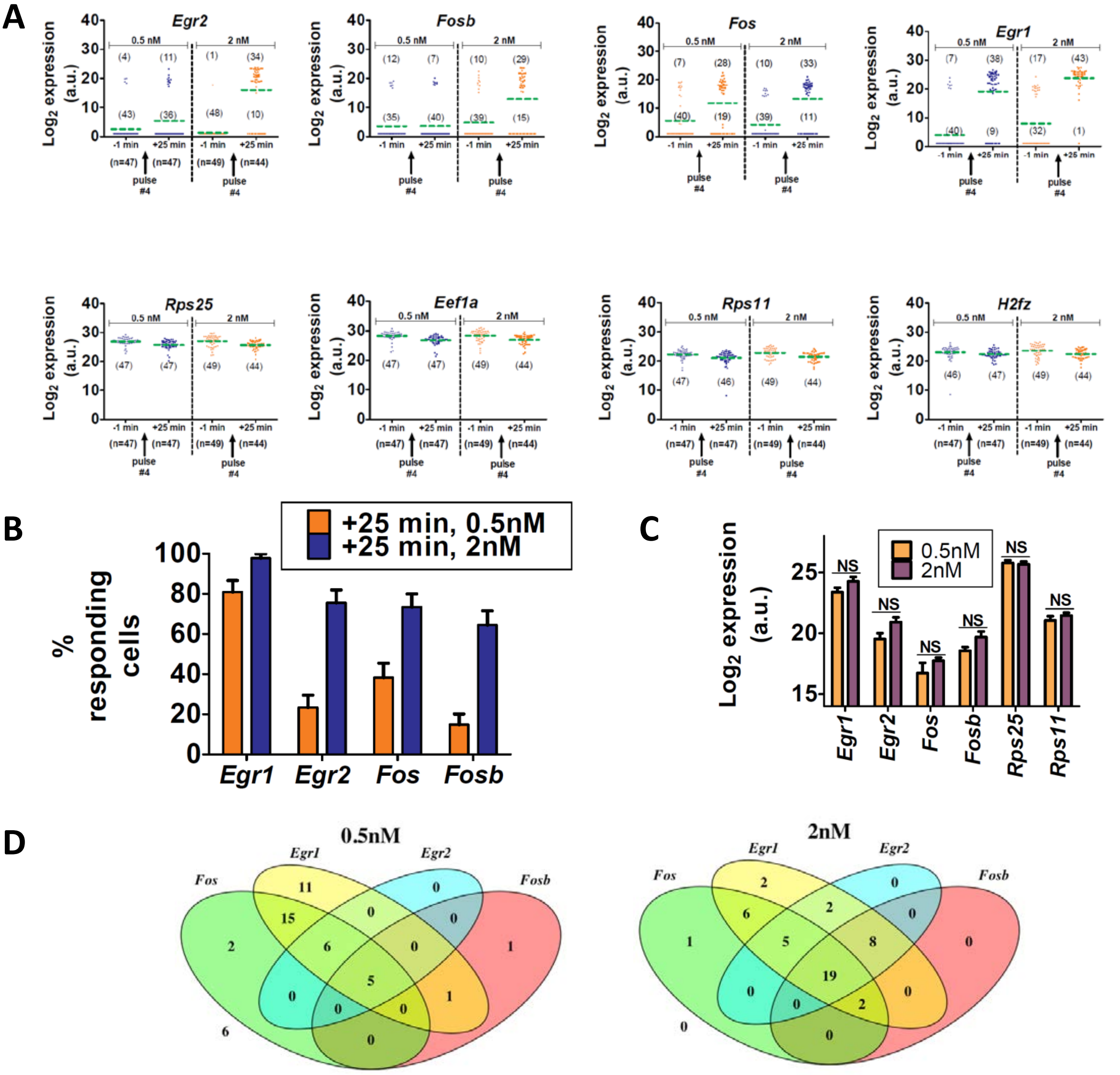

Supplementary Figure S15: Characterization of SC heterogeneity in the response of IEGs to different concentrations of GnRH (replicate experiment)

This is an independent experiment from the one shown in **Supplementary Fig. S13** (or **Fig. 5A**). Cells were exposed to either 0.5 or 2 nM GnRH and collected at either -1 min or +25 min relative to pulse #4. (**A**) Vertical scatter plots of IEG (*Top*) and housekeeping gene expression (*Bottom*) in SCs. In parentheses is indicated the number of gene-expressing (top) and non-gene expressing cells (bottom). Below each housekeeping gene plot is the total number of cells that were analyzed under each experimental condition. Each cell is represented either by a dot (-1 min) or a square (+25 min); the green dotted line signifies average gene expression in all analyzed cells under each experimental condition. (**B**) Bar graphs of the percentages of cells expressing a regulated gene (i.e. induced cells) at +25 min. Error bars are based on the binomial standard deviation on the number of gene-expressing cells. (**C**) Bar graphs of average gene expression in gene expressing (i.e. induced) cells at +25 min. Error bars represent standard deviation. ANOVA shows no significant differences. (**D**) Venn diagrams illustrating the overlap of *Fos*, *Egr1*, *Egr2*, and *Fosb* expression in all analyzed cells at +25 min.

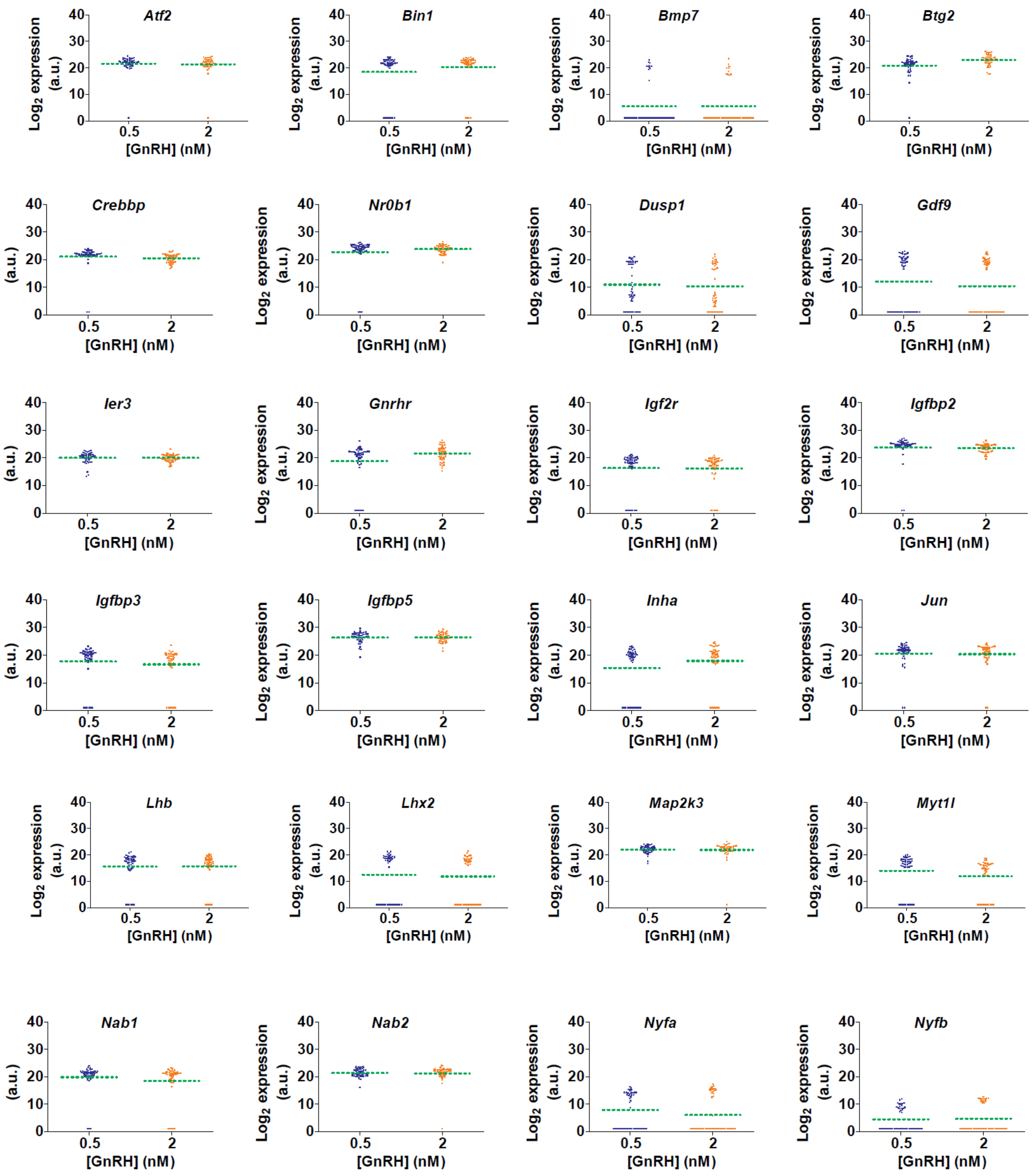

Supplementary Figure S16: Analysis of the SC response of 45 genes to different concentrations of GnRH (replicate experiment) (Part 1)

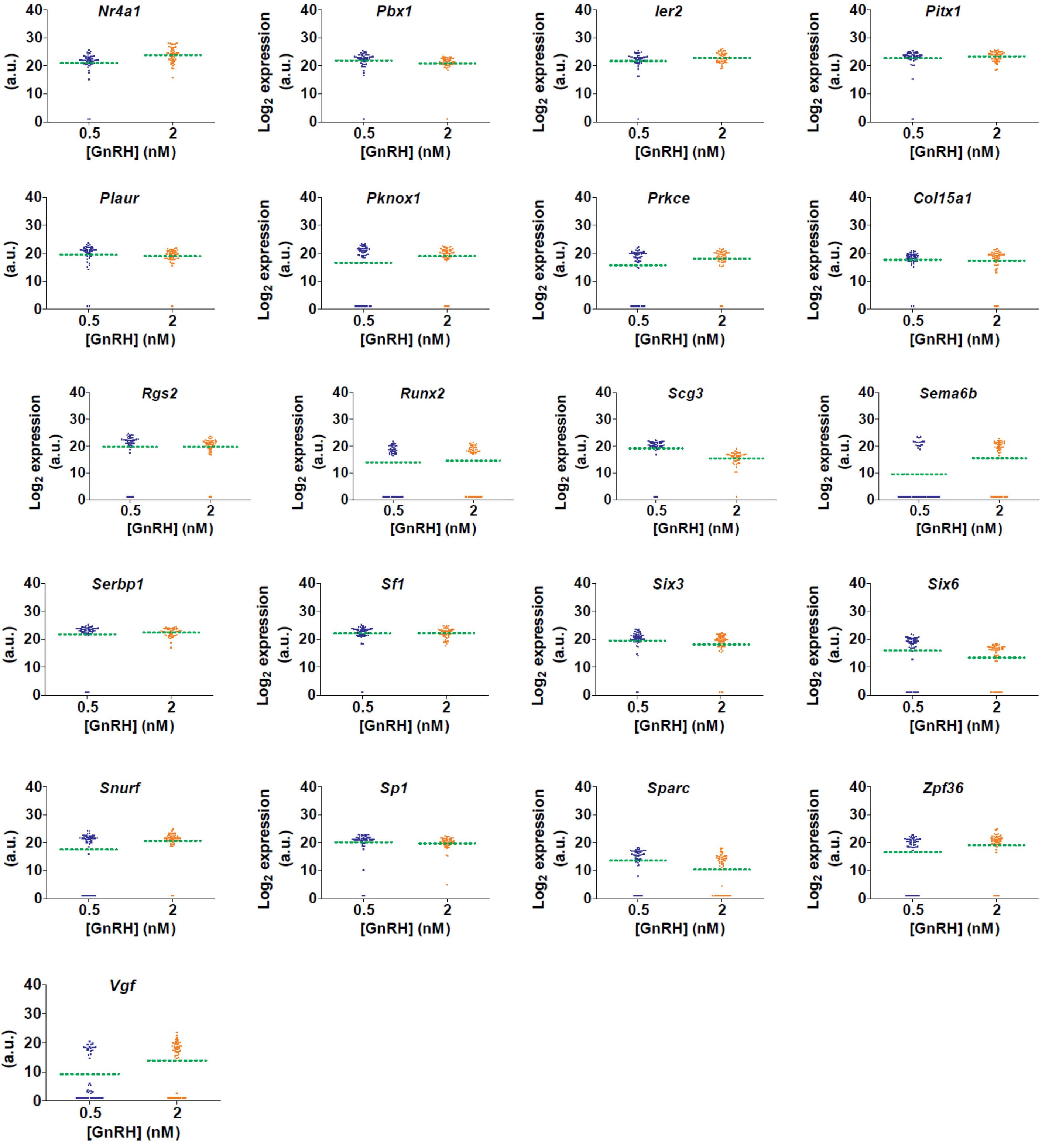

Supplementary Figure S16: Analysis of the SC response of 45 genes to different concentrations of GnRH (replicate experiment) (Part 2)

Vertical scatter plots of gene expression in SCs following exposure to increasing concentrations of GnRH and collection at +25 min relative to pulse #4 are shown for 45 genes. The green dotted line signifies average gene expression in all analyzed cells under each experimental condition. Data are from the same experiment as the one presented in **Supplementary Fig. S15**.
